# Ongoing Speciation and Gene Flow between Taxonomically Challenging *Trochulus* Species Complex (Gastropoda: Hygromiidae)

**DOI:** 10.1371/journal.pone.0170460

**Published:** 2017-01-20

**Authors:** Małgorzata Proćków, Tomasz Strzała, Elżbieta Kuźnik-Kowalska, Jarosław Proćków, Paweł Mackiewicz

**Affiliations:** 1 Museum of Natural History, University of Wrocław, Wrocław, Poland; 2 Department of Genetics, Faculty of Biology and Animal Science, Wrocław University of Environmental and Life Sciences, Wrocław, Poland; 3 Department of Invertebrate Systematics and Ecology, Institute of Biology, Wrocław University of Environmental and Life Sciences, Wrocław, Poland; 4 Department of Plant Biology, Institute of Biology, Wrocław University of Environmental and Life Sciences, Wrocław, Poland; 5 Department of Genomics, Faculty of Biotechnology, University of Wrocław, Wrocław, Poland; Universita degli Studi di Roma La Sapienza, ITALY

## Abstract

Geographical isolation, selection and genetic drift can cause the geographical diversification of populations and lead to speciation. Land snail species in the genus *Trochulus* show overlaps in geographical ranges as well as in morphology, but genetic data do not always support the species-level taxonomy based on morphological characters. Such a group offers an excellent opportunity to explore the processes involved. We have addressed the problem by determining the status of the restricted endemic *T*. *graminicola* within the larger context of *Trochulus* taxonomy. We used an integrated approach based on morphological features, ecological preferences and two molecular markers: mitochondrial COI sequences and microsatellites. Comparison of these results demonstrated: (i) conchological distinction of *T*. *striolatus* and *T*. *sericeus*; (ii) anatomical, ecological and genetic differentiation of *T*. *graminicola* and (iii) concordance between morphological characters and mtDNA markers in *T*. *striolatus*. Moreover, our data showed an intricate evolutionary history within the genus *Trochulus*, which can be best explained by: (i) recent or ongoing gene flow between taxa or (ii) their large ancestral polymorphism. Both of these hypotheses suggest that diversification within this group of snails has occurred relatively recently. The mismatches between species defined on morphology and on molecular genetics indicate the complexity of the processes involved in the diversification of this genus.

## Introduction

Determining the causes of difficulties in delimiting species in many taxa has proved hard. To explain the speciation process, different evolutionary mechanisms have been proposed such as: rapid and recent phylogenetic divergence, introgression, high phenotypic plasticity, ongoing differentiation of varieties and partial barriers to gene flow between ecotypes [1]. The uncertainty and controversy about species recognition are mostly related to four different issues: the assumed species concept, intrinsic properties of the species, speciation processes and methods used for inferring species boundaries [2].

Species delimitation remains a controversial issues in biological science [3]. A very popular method in the recognition of closely related species is DNA barcoding [4–6]. However, population genetic approaches based on microsatellites is a promising alternative. The usefulness of microsatellites has been frequently underlined in studies of demography and history of populations and determination of relationships between individuals in population, as well as in the estimation of effective population size and the magnitude and directionality of gene flow between populations (e.g. [7,8]). In these approaches, nuclear microsatellites allow the study of associations between morphological and molecular polymorphisms [1]. However, they have rarely been used for resolving boundaries between land snail species. The only study available to date on the delimitation of the *Trochulus sericeus/hispidus* complex is based on microsatellites [9]. Other studies were focused on the population genetic structure within species [10–13]. Moreover, recent investigations have demonstrated the limitations of DNA data, including barcoding attempts in highly diverse land snail species [14–16]. These studies showed low between species divergences, but high within species divergences; the barcoding gap between species is not clearly defined. However, specialized methods, e.g. the Automatic Barcoding Gap Detection (ABGD) method and the General Mixed Yule-Coalescent model (GMYC) [17–20] can help make the delimitation of species more clear.

The difficulties in defining the number and boundaries of species in the genus *Trochulus* Chemnitz, 1786 has provoked continuous debate [21]. Great morphological variability and a multiplicity of forms led some authors to describe many species, e.g. Locard [22,23] listed 55 species in France, while Germain [24] distinguished only a few. The research history of the genus was discussed in detail by [25]. Recent studies on *Trochulus* species have been based on both morphological and genetic data [16,26–29]. They revealed very complicated relationships among the taxa, raising questions about the status of forms resembling *T*. *hispidus* (Linnaeus, 1758), such as *T*. *sericeus* (Draparnaud, 1801), *T*. *plebeius* (Draparnaud, 1805) and *T*. *coelomphala* (Locard, 1888). At the same time, an increasing number of studies indicated the existence of cryptic species [30], also within *Trochulus* [9,31]. Another poorly known species that is involved in this taxonomic Gordian knot is *T*. *graminicola* (Falkner, 1973).

*Trochulus graminicola* is an endemic species known only from south-western Germany. Its type locality, Eichberg near Blumberg in Baden-Württemberg, was precisely described by Falkner [32]. The habitat of *T*. *graminicola* is unique for the genus, as it lives in grassy screes in sparse montane forests on steep slopes with pine, spruce and juniper (Calamagrostio variae-Pinetum association). Both juveniles and adults stay firmly attached to grass blades, especially to their underside. While feeding, they choose wider, damaged blades. Snails are not found directly on the ground or on shrubs [32]. In the original description, shells were characterized as strongly flattened, usually twice as broad as high, with a nearly flat spire and usually 5.7 whorls. Their aperture is oblique and aperture margin reflected with a white lip. The umbilicus is wide and oval. Adults are hairless, while hairs of juveniles are short and weak. The shell is dark horny to reddish-brown, irregularly prominently striated above and smooth, distinctly convex below and shiny, sometimes with a light band on the body whorl [32]. It differs from a similar flat form of *T*. *hispidus* that is sometimes regarded as *T*. *coelomphala* by its larger dimensions, even flatter spire, oblique aperture, stronger striation and chestnut brown colour. The genital morphology of *T*. *graminicola* is also distinctive and concerns a longer upper vagina [32]. It can be distinguished from flat forms of *T*. *montanus* by a slender reproductive system with an elongated oval bursa copulatrix and widely open umbilicus, which makes all previous whorls visible [32]. Despite this quite precise description, identification of the species remains problematic [33]. The problems result from the considerable similarity in shell morphology among *Trochulus* species and a significant influence of the environment on variation in their shell size and shape, which have led to the assignment of extremely distinct morphological forms to the same species [29]. Hence, morphological characters alone may be an unreliable means of distinguishing species.

Using both molecular (based on mtDNA and microsatellites) and morphological (based on shell and genitalia) methods, we revise the taxonomic status of *T*. *graminicola* and assess whether it is a true species according to typological and phylogenetic species concepts. Combining molecular, morphological and ecological data, we place this species into the larger context of *Trochulus* taxonomy and test its relationship with closely related and morphologically similar taxa, i.e. *T*. *coelomphala*, *T*. *hispidus*, *T*. *sericeus* and *T*. *striolatus* inhabiting adjacent areas. Additionally, we analyse several samples collected from more distant regions (Poland and Great Britain). Finally, we compare the morphological recognition of taxa with their clustering in phylogenetic trees based on mtDNA. Since the morphological identification of these species is not always certain [28,31,34], we provisionally assumed their classification provided in malacological literature based on the morphological traits of shell and genitalia [21,32,35–38] to check the consistency of morphological and molecular approaches. Moreover, we examine the correspondence between mitochondrial and microsatellite markers to search for possible cases of introgression.

## Materials and Methods

### Sample collection, niche description, shell and genital morphometry

**Fig 1:**
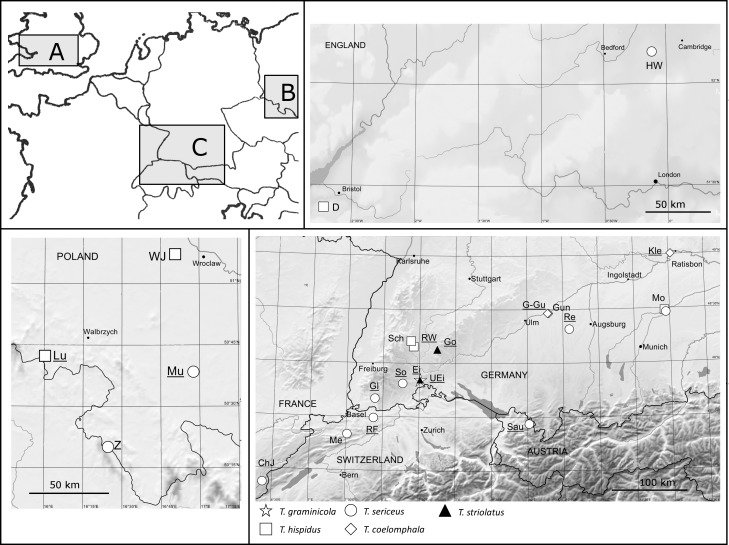
Geographical locations of the populations sampled. (A) Great Britain, (B) Poland and (C) Germany, France, Switzerland and Austria. Populations analysed with both COI sequences and microsatellites are underlined.

The complete list of sample sites and specimens, collected in three seasons between July and August 2011–2013 or analysed elsewhere [28,29], is shown in Table 1 and Fig 1. All the material is deposited in the Museum of Natural History in Wrocław, Poland. *Trochulus graminicola* was found only at the type locality with a limited number of specimens, and most of the other *Trochulus* populations came from adjacent areas (Fig 1). Snails from Zieleniec and Hayley Wood, determined as *T*. *plebeius* by Wiktor [39] and Paul [40], were assigned by us to *T*. *sericeus* and we use the name *T*. *plebeius/sericeus* throughout the paper. We use this terminology because the name *T*. *plebeius* should rather be reserved for similar forms from the French and Swiss Jura [29]. In addition, vegetation and dominant plant species were recorded at each site and habitat specified (Table 1). For precise niche descriptions of *T*. *graminicola*, plant nomenclature follows Oberdorfer [41].

**Table 1:** List of localities and individuals included in the present study.

Abbreviation: location	Habitat	Altitude a.s.l. (m)	Coordinates	Voucher number of reference collection (NCBI Accession Number–COI)	Voucher number of reference collection–shell	Voucher number of reference collection–genitalia
*T*. *graminicola*						
Ei: Eichberg, Baden-Württemberg, Germany (G)	grass in sparse forest with pine, spruce and juniper (Calamagrostio variae-Pinetum ass.)	829	47°50’45.8” N 08°30’46.1” E	Ei_1 (KU556336)	Ei_6	Ei_6
				Ei_2 (KU556353)		
				Ei_3 (KU556340)		
				Ei_4 (KU556337)		
				Ei_5 (KU556338)		
				Ei_6 (KU556339)		
Ei: Eichberg, Baden-Württemberg, Germany (G)			SMF-117324/9	-	Ei_7–Ei_14*	-
Ei: Eichberg, Baden-Württemberg, Germany (G)			types	-	Ei_15–Ei_24^#^	Ei_15–Ei_22^#^
*T*. *coelomphala*						
G-Gu: Günzburg-Reisensburg, Bavaria, Germany (G)	small-leaved lime forest with ash	436	48°27’56.8” N 10°18’05.9” E	^a)^G-Gu_8 (JX475089)	G-Gu_1– G-Gu_8	G-Gu_7
				G-Gu_9 (JX475090)		G-Gu_8
				G-Gu_10 (JX475091)		
Gun: Günzburg, Bavaria, Germany (G)	mixed ruderal–meadow riverbank vegetation	442	48°27’56.1” N 10°17’08.7” E	Gun_1 (KU556341)	Gun_1–Gun_31	Gun_1–Gun_15
				Gun_3 (KU556345)		
				Gun_4 (KU556343)		
				Gun_12 (KU556344)		
Kle: Kleinprüfening, Bavaria, Germany (G)	glade surrounded by *Salix* sp., *Prunus padus*, *Cornus* sp.	326	49°00’18.9” N 12°01’50.3” E	Kle_1 (KU5563550	Kle_1–Kle_12	Kle_1
				Kle_2 (KU556388)		
				Kle_3 (KU556356)		
				Kle_4 (KU556385)		
				Kle_5 (KU556387)		
				Kle_8 (KU556386)		
*T*. *hispidus*						
RW: Ruine Waldau, Baden-Württemberg, Germany (G)	ruderal vegetation along walls of castle ruin	751	48°09’23.0” N 08°24’50.8” E	RW_1 (KU556347)	RW_1–RW_14	RW_1– RW_6
				RW_2 (KU556348)		RW_9
				RW_3 (KU556349)		
				RW_4 (KU556350)		
				RW_5 (KU556352)		
				RW_9 (KU556351)		
Lu: Lubawka, Lower Silesia, Poland (P)	open, human-affected nettle (*Urtica dioica*) patch	420	50°42’19.2” N 16°00’09.8” E	Lu_1 (KU556359)	Lu_1–Lu_65	Lu_1–Lu_4
				Lu_2 (KU556334)		
				Lu_3 (KU556333)		
WJ: Wrocław-Jarnołtów, Lower Silesia, Poland (P)	open, human-affected nettle (*Urtica dioica*) patch	130	51°07’16.9” N 16°50’38.7” E	^a)^WJ_1 (JX475096)	WJ_2–WJ_31	WJ_31–WJ_33
				WJ_2 (JX475097)		WJ_35
				WJ_3 (JX475098)		WJ_36
D: Downside, North Somerset, England (UK)	house garden	160	51°23’28.4” N 02°43’06.9” W	^a)^D_1 (JX475050)	D_1–D_9	D_2
				D_2 (JX475051)		D_3
				D_3 (JX475052)		D_5
				D_4 (JX475053)		
Mo: Moosburg a.d. Isar, Bavaria, Germany (G)	riverbank vegetation	376	48°28’57.0” N 11°55’45.5” E	^a)^Mo_1 (JX475092)	Mo_2–Mo_5	Mo_3–Mo_5
				Mo_2 (JX475093)		
				Mo_4 (JX475094)		
Sch: Schramberg, Schwarzwald, Germany (G)	ruderal vegetation along footpath	508	48°12’26.5” N 08°22’46.0” E	^a)^Sch_4 (JX475095)	Sch_1–Sch_2	Sch_6–Sch_8
					Sch_4–Sch_8	
*T*. *sericeus*						
Sau: Sausteig, Vorarlberg, Austria (A)	vegetation along roadside shaded by alder (*Alnus viridis*)	994	47°26’21.2” N 10°01’10.1” E	Sau_1 (KU556378)	Sau_1–Sau_4	Sau_1–Sau_2
				Sau_2 (KU556379)		
				Sau_3 (KU556357)		
				Sau_4 (KU556358)		
Re: Reischenau, Bavaria, Germany (G)	meadow vegetation along drainage ditch	468	48°19’04.2” N 10°35’22.1” E	Re_1 (KU556360)	Re_1–Re_13	Re_1–Re_10
				Re_2 (KU556354)		
				Re_3 (KU556335)		
				Re_4 (KU556383)		
				Re_5 (KU556384)		
So: Sommerau, Baden-Württemberg, Germany (G)	ruderal riverbank vegetation	878	47°48’46.7” N 08°16’08.4” E	So_1 (KU556373)	So_1–So_10	So_1–So_6
				So_2 (KU556376)		So_8
				So_3 (KU556374)		So_9
				So_4 (KU556375)		
Gl: Glashütten, Baden-Württemberg, Germany (G)	roadside, edge of fir forest	563	47°40’14.9” N 07°53’16.8” E	Gl_1 (KU556380)	Gl_1–Gl_3	Gl_1
				Gl_2 (KU556381)		
				Gl_3 (KU556382)		
RF: Ruine Farnsburg, Baselland, Switzerland (S)	limestone rocks	721	47°29’30.6” N 07°52’12.4” E	RF_1 (KU556346)	RF_1–RF_35	RF_1–RF_5
				RF_3 (KU556377)		
				RF_4 (KU556342)		
Mu: Muszkowice, Lower Silesia, Poland (P)	natural ash forest	219	50°38.458’ N 16°57.050’ E	Mu_1 (KU556369)	Mu_1–Mu_23	Mu_1–Mu_7
				Mu_2 (KU556372)		
				Mu_3 (KU556370)		
				Mu_4 (KU556371)		
Z: Zieleniec, Sudetes, Poland (P)	natural ash forest mainly with *Petasites* sp. div.	686	50°20’07.7” N 16°24’34.6” E	^a)^Z_3 (JX475099)	Z_1–Z_32	Z_1–Z_5
				Z_5 (JX475100)		
HW: Hayley Wood, Cambridgeshire, England (GB)	wood with pedunculate oak and regularly coppiced understory of hazel	84	52°09’31.0” N 0°06’54.9” W	^a)^HW_3 (JX475054)	HW_1–HW_31	HW_5
				HW_4 (JX475055)		HW_6
				HW_5 (JX475056)		HW_16
				HW_7 (JX475057)		HW_19
						HW_20
ChJ: Château de Joux, Doubs, France (F)	open limestone rocks	911	46°52’19.8” N 06°22’22.5” E	^a)^ChJ_39 (KF812936)	ChJ_38	-
				ChJ_40 (KF812937)		
Me: Mervelier, Jura, Switzerland (S)	shaded limestone rocks	620	47°20’09.0” N 07°30’50.5” E	^a)^Me_4 (KF812938)	Me_4	-
				Me_6 (KF812939)		
				Me_7 (KF812940)		
Mo: Moosburg a.d. Isar, Bavaria, Germany (G)	riparian forest	382	48°27’53.3” N 11°56’50.5” E	^a)^Mo_46 (KF813000)	Mo_44	-
				Mo_47 (KF813001)	Mo_50	
				Mo_48 (KF813002)		
				Mo_49 (KF813003)		
				Mo_45 (KF813004)		
*T*. *striolatus*						
UEi: Untere Eichberg, Baden-Württemberg, Germany (G)	mixed forest	692	47°50’49.9” N 08°30’30.0” E	UEi_2 (KU556365)	UEi_2–UEi_31	UEi_2–UEi_7
				UEi_3 (KU556361)		UEi_12
				UEi_4 (KU556363)		UEi_17
				UEi_5 (KU556362)		UEi_20–UEi_22
				UEi_7 (KU556364)		UEi_24
						UEi_28
Go: Gosheim, Baden-Württemberg, Germany (G)	natural ash and maple forest	851	48°07’41.1” N 08°44’48.2” E	Go_1 (KU556366)	Go_1–Go_7	Go_1–Go_5
				Go_2 (KU556367)		
				Go_3 (KU556368)		
Ei: Eichberg, Baden-Württemberg, Germany (G)	sparse forest with pine, spruce and juniper (Calamagrostio variae-Pinetum ass.)	829	47°50’45.8” N 08°30’46.1” E	-	Ei_25–Ei_26	-
Ei: Eichberg, Baden-Württemberg, Germany (G)			SMF-337318/3	-	Ei_27–Ei_29*	-

Abbreviations

* specimens from Senckenberg Museum collections in Frankfurt (SMF)

^#^ measurements of type specimens from Falkner [32]

^a)^ sequences from GenBank analysed by Proćków et al. [28,29].

All 397 shells were measured in standardized views [25] by the same person (MP) with a graduated eyepiece in a stereomicroscope and an accuracy of 0.1 mm. Since the systematic measurement error (with 1% error probability) does not compromise results [26], one measure of each variable on each specimen was considered sufficient. Only adult individuals (as defined in [28]) were considered. Finally, basic statistical parameters were calculated.

Each individual was measured from the side perspective for shell height (H), shell width (W), aperture height (h), aperture width (w) and body whorl height (bwH). From underneath, umbilicus major diameter (U) (i.e. the longest diameter parallel to the shell diameter, D), umbilicus minor diameter (u) (i.e. perpendicular to the umbilicus major diameter) and shell diameter (D) were taken. Finally, the number of whorls (whl) was counted according to Ehrmann’s method [42]. In addition, the following coefficients of shell proportions were calculated: height/width ratio (H/W), relative height of body whorl (bwH/H), relative umbilicus diameter (U/D) (umbilicus major diameter/shell diameter ratio), ratio of umbilicus minor to its major diameter (u/U) and height aperture/width aperture ratio (h/w).

For genital morphological examinations, 114 mature snails (Table 1) were dissected and seven measurements of genitalia were taken: the length of flagellum (fl), epiphallus (ep), penis (p), bursa copulatrix (sl), bursa duct (sd), upper vagina (uv) (i.e. distance between outlet of mucous glands and tips of inner dart sacs) and the width of bursa copulatrix (sw). The relative length of inner to outer dart sacs (is/os) was also recorded by measuring the distance between the tips of inner and outer dart sacs. Then the following proportions were calculated: flagellum/epiphallus (fl/ep), epiphallus/penis (ep/p), bursa duct length/bursa length (sd/sl) and bursa width/length (sw/sl), and included in statistical analyses.

Measurements were used in canonical discriminant analysis (CDA). To reduce the number of highly correlated predictors, we performed principal component analysis (PCA) using a correlation matrix and used the first two principal components in further studies. We also computed pairwise Pearson correlation coefficients involving all variables and next reduced the number of the most correlated parameters. Based on these results, six shell variables (W, H, D, w, U and u) were removed because they were characterized by the largest mean absolute correlation. We assumed a pairwise absolute correlation cut-off of 0.9 and re-evaluated the average correlations at each step of the elimination. These procedures allowed us to reduce the number of redundant variables and leave the best predictors describing differences between the examined morphospecies. Variables contributing most to CDA, i.e. the most promising in species distinction, were further used in Kruskal–Wallis non-parametric analysis of variance (ANOVA). For statistical analysis of the data, Statistica 10 software (StatSoft, Inc. 1984–2011) was used. The elimination of correlated parameters was made by findCorrelation from the caret package in R [43].

### Ethics statement

Samples were taken only on communal lands. No specific permission was required for crossing these areas or carrying out snail surveys. The target species are not protected by any national law or local regulations.

### Genetic analysis

#### DNA extraction, PCR amplification and sequencing

A snail foot fragment or, in the case of juvenile specimens, the entire animals (both preserved in ethanol) were used for DNA extraction. Total genomic DNA was isolated from 56 individuals with Sherlock AX (A&A Biotechnology, Gdynia, Poland) according to the manual. Quality and quantity of isolated DNA was assessed in gel electrophoresis (1% agarose gel). The analysed DNA fragment of cytochrome c oxidase subunit I (COI) was amplified with primers according to PCR conditions described in [29]. PCR product was sequenced in both directions with an ABI 3730 sequencer. Furthermore, six microsatellite loci were amplified: TROA3, TROA4, TROA5, TROA6, TROB5 and TROB105. All microsatellite loci, their length and repeat size along with amplification conditions were previously described by Dépraz et al. [44]. Amplification was performed in three multiplex reactions (two loci in each multiplex) with a Qiagen Multiplex PCR Kit (in 10 μl volume) according to the producer’s manual. To minimize genotyping errors we used aerosol-resistant filter tips, exposed all equipment to UV radiation prior to use and added negative controls to all reactions. Lengths of the microsatellite products were read using an ABI 3730 sequencer (Wyzer Biosciences Inc., MA, USA) and GeneMarker 2.4.2 software (SoftGenetics, www.softgenetics.com). All nucleotide sequences reported in this study have been deposited in GenBank with accession numbers KU556333–KU556388.

#### Population genetic data analysis

Since we were interested in genetic structure of analysed individuals, we calculated the probability of individual membership to distinct genetic clusters, using the Bayesian approach, as implemented in Structure 2.3.4 [45–47]. We used correlated allele frequencies with an admixture model without informing the former about sample localities. The calculations run for 1,000,000 replicates with burn-in in the 200,000 step. After that, we analysed K clusters from 1 to 20 and ten replicates for each K. The Delta K method [48] along with standard prediction of K based on the plotted mean ln probability of K (L(K)) were used to find the most probable number of distinct genetic clusters. Both plots (Delta K and L(K)) were calculated using Structure Harvester [49]. Clumpak package [50] with implemented Clumpp [51] and Distruct [52] software were used to estimate pairwise similarities between runs and to visualize the most probable clustering scheme.

#### Phylogenetic inference

Phylogenetic analyses were based on two data sets created by 98 aligned sequences of mitochondrial COI. The first set (called long) included 655 positions, whereas the second one (short) contained 561 positions, after exclusion of alignment positions that contained incomplete terminal sequence in at least one taxon. Sequences newly obtained in this study were combined with 39 previously published sequences [16,29] collected from BLAST searches of the GenBank database. Sequences of three representatives from other Hygromiidae: *Candidula unifasciata* (Poiret, 1801), *Lindholmiola girva* (Frivaldszky, 1835) and *Kovacsia kovacsi* (Varga & Pintér, 1972) were used as an outgroup (accession numbers JX911297, EU182448 and EU182471, respectively). To infer phylogenetic trees, we applied four approaches: Bayesian in MrBayes 3.2.3 [53] and PhyloBayes MPI 1.5a [54] and maximum likelihood in TreeFinder (partitioned model) [55] and PAUP* 4.0b (not-partitioned model) [56] as well as maximum parsimony and neighbour-joining methods in PAUP*.

In MrBayes analyses, we assumed three separate mixed models for three codon positions to sample appropriate models across the substitution model space in the Bayesian MCMC analysis itself, avoiding the need for *a priori* model testing [57]. In addition, we applied gamma-distributed rate variation across sites with five discrete rate categories for the first and third codon positions as suggested by jModelTest 2.1 [58,59]. In the analyses, two independent runs starting from random trees, each using four Markov chains, were applied. Trees were sampled every 100 generations for 10,000,000 generations. In the final analysis, we selected trees from the last 3,779,000 (for the long dataset) and 4,271,000 (for the short dataset) generations that reached the stationary phase and convergence (i.e. the standard deviation of split frequencies stabilized and was lower than the proposed threshold of 0.01). In PhyloBayes, we used the CAT-GTR model with rate variation across sites modelled by five discrete rate categories of gamma distribution. The number of components, weights and profiles of the model were inferred from the data. Two independent Markov chains were run for 100,000 generations in each of these analyses. The last 25,000 (for the long dataset) and 5000 (for the short dataset) trees from each chain were collected to compute posterior consensus trees after reaching convergence, when the largest discrepancy observed across all bipartitions (maxdiff) was below the recommended 0.1.

In TreeFinder tree reconstruction, we applied search depth level 2 and separate substitution models for three codon positions as suggested by this program’s Propose Model module according to AIC, AICc, BIC and HQ criteria, for the long dataset: J2+Γ(5), HKY{3,1,1,1,1,3}+ Γ(5), TVM+Γ(5) and for the short dataset: J2+Γ(5), J3+I, TVM+Γ(5). In the case of maximum likelihood and parsimony approaches in PAUP, the final tree was searched from ten starting trees obtained by stepwise and random sequence addition followed by the tree bisection and reconnection (TBR) branch-swapping algorithm. The maximum likelihood and neighbour-joining trees were calculated using the best-fit substitution model TPMuf+I+Γ(5), as found in jModelTest among 1624 candidate models considering all four criteria: AIC, AICc, BIC and DT. Non-parametric bootstrap analyses were performed for two datasets using 1000 replicates for neighbour joining, maximum likelihood and parsimony methods. In addition to that, the number of approaches supporting a given node was calculated using consent from PHYLIP package 3.69 [60] and superimposed on MrBayes trees.

Intra- and interclade diversity was calculated as *p*-distance (the proportion of nucleotide sites at which two compared sequences are different) for the long dataset with the pairwise deletion option using MEGA 6.05 package [61]. Its variance was computed by the bootstrap method assuming 1000 replicates. Three outgroup sequences were excluded in this calculation.

### Procedures of species delimitation

For the delimitation of species, we used the long alignment of COI sequences and applied the Automatic Barcoding Gap Detection (ABGD) method [62] as well as the General Mixed Yule-Coalescent model (GMYC) [63,64]. ABGD was carried out via a web interface (http://www.abi.snv.jussieu.fr/public/abgd/abgdweb.html) using a distance matrix obtained from the phylogenetic tree inferred in MrBayes. GMYC analyses were carried out in the R environment [43] using the Splits package [65]. We applied both single [63] and multiple threshold models [64]. The input tree was obtained by the conversion of the MrBayes tree to the ultrametric one using the chronopl command from the Analyses of Phylogenetics and Evolution (Ape) package in R [66], which implements the penalized likelihood method [67].

### Testing consistency of morphological, molecular and geographical data

To assess consistency of taxa delimitation by different approaches, we applied a likelihood ratio G-test of independence assuming Williams’ continuity correction. We tested, in pairwise comparisons, independence of an individual’s assignment to species, geographical location, morphological groups (assuming *T*. *hispidus* and *T*. *coelomphala* as one group), clades based on mitochondrial COI phylogeny and microsatellite clusters. In the last case, we considered both 12 microsatellite groupings of individuals (four clear clusters and eight mixed assignments of individuals) and five microsatellite groups, in which the eight mixed groups were considered as one cluster in addition to the four clear clusters.

Moreover, we used a Mantel test assuming 10,000 permutations to estimate the significance of correlation in pairwise comparisons of matrices including: Euclidean distances between snail samples for all shell and genitalia measurements, geographical distance and absolute differences between altitude of sites and where samples were found as well as the number of base differences per site between COI sequence and distances between microsatellite data, calculated according to the method of Nei et al. [68]. The metric features were subjected to minimum–maximum normalization before the calculation of distances. The geographical distances were calculated as orthodromic distances based on geographical latitudes and longitudes of the sites assuming a mean earth radius of 6371 km.

G-test was carried out by the likelihood.test function and Mantel test by the mantel.rtest function as implemented in R package [43]. The Benjamini–Hochberg method for correction of the obtained *p*-values was applied to control the false discovery rate in the two approaches.

## Results

### Morphometric data

**Fig 2:**
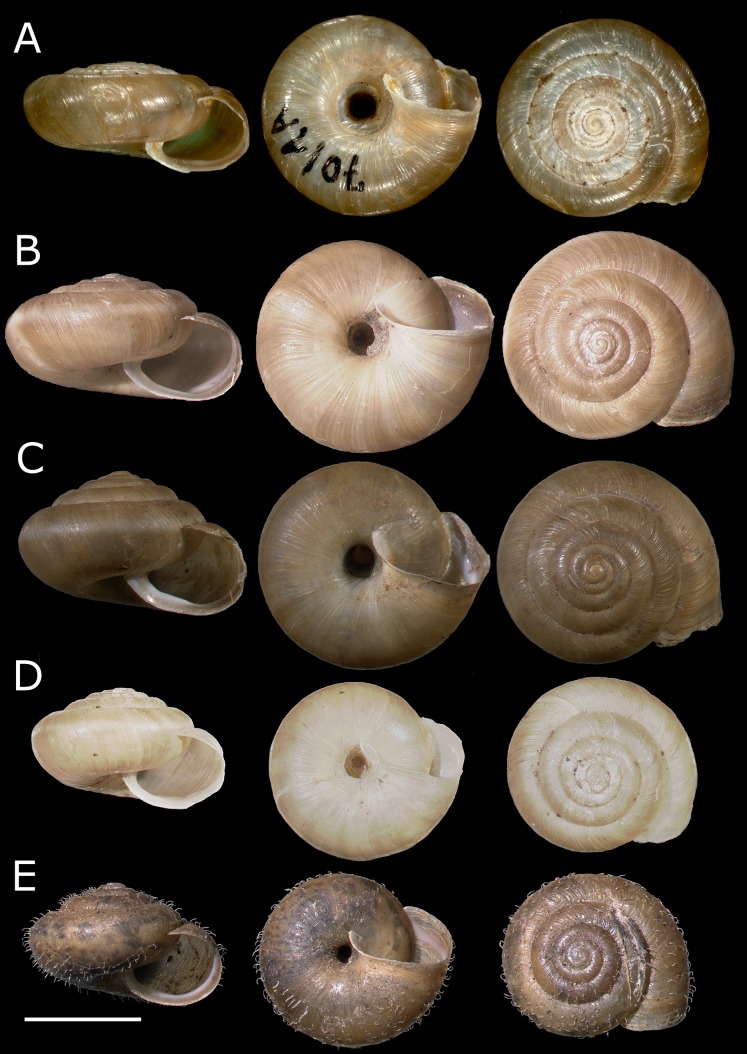
**(A) *Trochulus graminicola*, paratype specimen (SMF 228814) from Eichberg (photo: S. Hof, courtesy of R. Janssen, Senckenberg Research Institute, Frankfurt M.). (B) *T*. *striolatus*, specimen from Untere Eichberg. (C) *T*. *striolatus*, specimen from Gosheim. (D) *T*. *sericeus*, specimen from Sausteig. (E) *T*. *sericeus*, specimen from Glashütten.** Scale bar: 5 mm.

**Fig 3:**
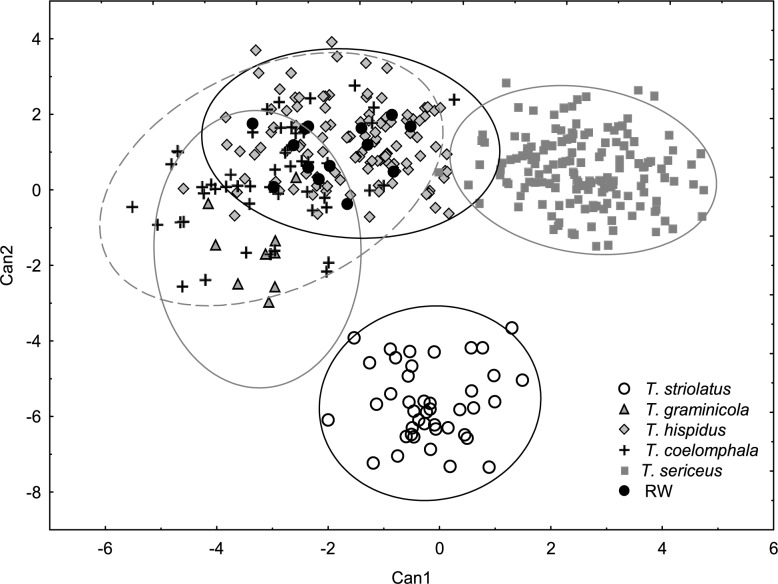
Canonical discriminant analysis based on shell measurements of *Trochulus* taxa and Ruine Waldau population (RW). Wilks’ lambda = 0.02005, *F*_60,1778_ = 38.669, *p* < 0.00001.

**Fig 4:**
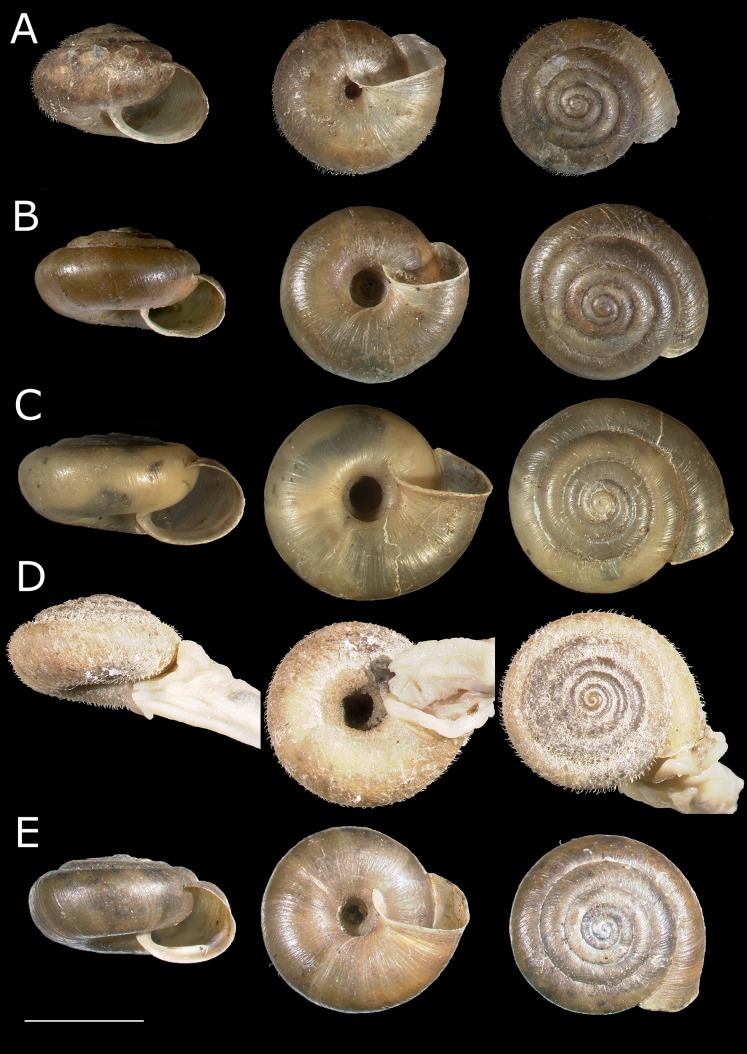
**(A) *Trochulus sericeus*, specimen from Hayley Wood. (B) *T*. *hispidus*, specimen from Downside. (C) *T*. *coelomphala*, specimen from Günzburg. (D) *T*. *coelomphala*, specimen from Kleinprüfening. (E) *T*. *hispidus*, specimen from Ruine Waldau.** Scale bar: 5 mm.

**Fig 5:**
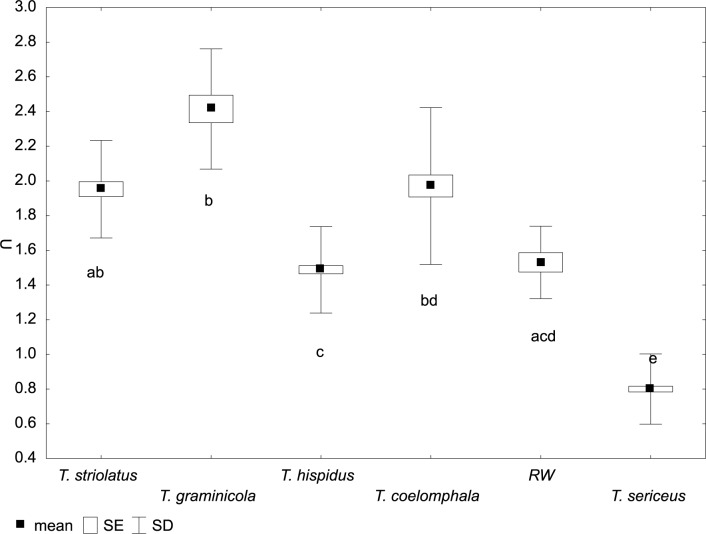
Variation of umbilicus diameter (U) in *Trochulus* taxa and Ruine Waldau population (RW). Letters a–e indicate significant differences determined by the Kruskal–Wallis test (*p* < 0.05).

**Fig 6:**
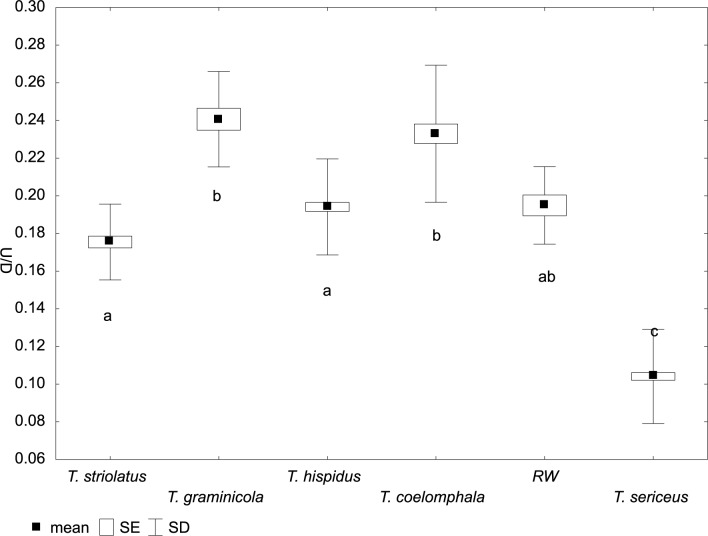
Variation of relative umbilicus diameter (U/D) in *Trochulus* taxa and Ruine Waldau population (RW). Letters a–c indicate significant differences determined by the Kruskal–Wallis test (*p* < 0.05).

The CDA of shell morphology correctly assigned individuals to species in 90.9% of cases (Table 2) and indicated that 93.7% of the variation could be explained by two discriminant functions. The first (Can1) and the second (Can2) functions detected 51.4% and 42.3% of variation, respectively. The first function distinguished individuals by the relative umbilicus diameter (U/D) and the second by the body whorl height (bwH) (Table 3). Thus, morphometric analysis sustained the recognition of three distinct morphological groups of *Trochulus* occurring in sympatry in the study area (Fig 1). They may be attributed to three morphologically recognized species, i.e. *T*. *graminicola*, *T*. *striolatus* and *T*. *sericeus* (Figs 2 and 3). However, only *T*. *striolatus* was separated as a distinct set. Overlapping subsets were formed by *T*. *hispidus* and *T*. *coelomphala*, whose shells strongly resemble each other (Fig 4). The first of these two species also demonstrated a moderate overlap of variation ranges with *T*. *sericeus* and *T*. *graminicola*, while *T*. *coelomphala* formed an overlapping subset with *T*. *graminicola*. *Trochulus graminicola*, *T*. *striolatus* and *T*. *coelomphala* were well separated from *T*. *sericeus* on the basis of broader umbilicus (mean > 1.5 mm), revealing statistically significant differences (Kruskal–Wallis test: *p* < 0.001; Fig 5). U/D may be a discriminant character for *T*. *graminicola* compared to *T*. *striolatus*, *T*. *hispidus* and *T*. *sericeus* because the differences were statistically significant (Kruskal–Wallis test: *p* < 0.05). Similarly, *T*. *hispidus* and *T*. *sericeus* could also be distinguished from each other to some extent based on the latter character (Kruskal–Wallis test: *p* < 0.001; Fig 6). The values of U/D ranged from 0.14 to 0.27 (mean = 0.19) in *T*. *hispidus* and from 0.05 to 0.18 (mean = 0.10) in *T*. *sericeus*. Since the specimens from Ruine Waldau (RW) were difficult to assign to a certain species, they were separately studied in the CDA. Subsequently, all individuals were placed in the position between *T*. *hispidus* and *T*. *coelomphala* (Figs 3 and 4). Statistically significant differences of the RW population were recorded for U and U/D but only with respect to *T*. *sericeus* (Figs 5 and 6). The shells of RW specimens were correctly classified in 42.9% of cases (Table 2).

**Table 2:** CDA result of *a posteriori* classification of shell morphology in *Trochulus* taxa and Ruine Waldau population (RW).

	A priori group						
A posteriori group	*T*. *striolatus*	*T*. *graminicola*	*T*. *hispidus*	*T*. *coelomphala*	*T*. *sericeus*	RW	Percentage of correctly classified
*T*. *striolatus*	42	0	0	0	0	0	100.0
*T*. *graminicola*	0	7	0	2	0	0	77.8
*T*. *hispidus*	0	0	108	5	0	2	93.9
*T*. *coelomphala*	0	3	10	35	0	3	68.6
*T*. *sericeus*	0	0	2	0	153	0	98.7
RW	0	0	2	6	0	6	42.9
Total	42	10	122	48	153	11	90.9

**Table 3:** Canonical coefficients of discriminant analysis performed on shell measurements.

Variable	Standardized canonical discriminant function coefficients	
	Can1	Can2
U/D	**-0.967**	0.006
bwH	0.399	**-1.168**
H/W	0.086	0.708
u/U	-0.515	-0.080
bwH/H	0.007	0.437
whl	-0.155	0.371
h	-0.322	-0.174
h/w	0.077	-0.227
Eigenvalue	5.103	4.204
Cum. Prop. (%)	51.4	93.7

The highest values are in **bold**. For abbreviations, see Materials and Methods.

**Fig 7:**
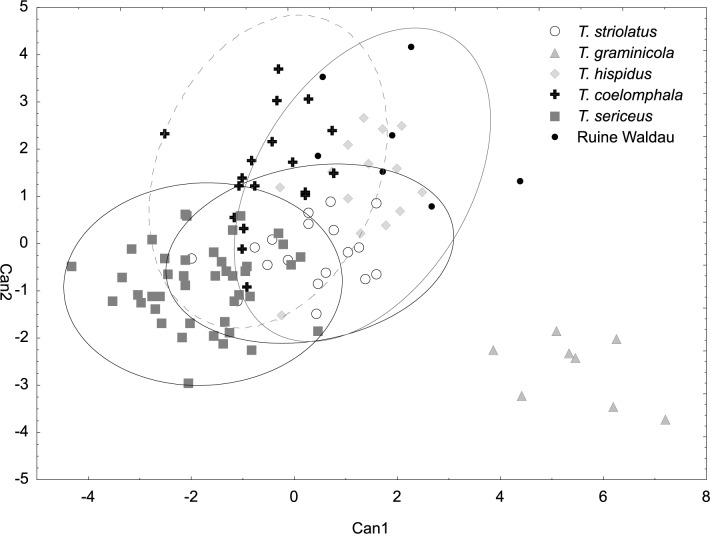
Canonical discriminant analysis based on genital measurements of *Trochulus* taxa and Ruine Waldau population (RW). Wilks’ lambda = 0.02017, *F*_55,434_ = 10.450, *p* < 0.00001.

**Fig 8:**
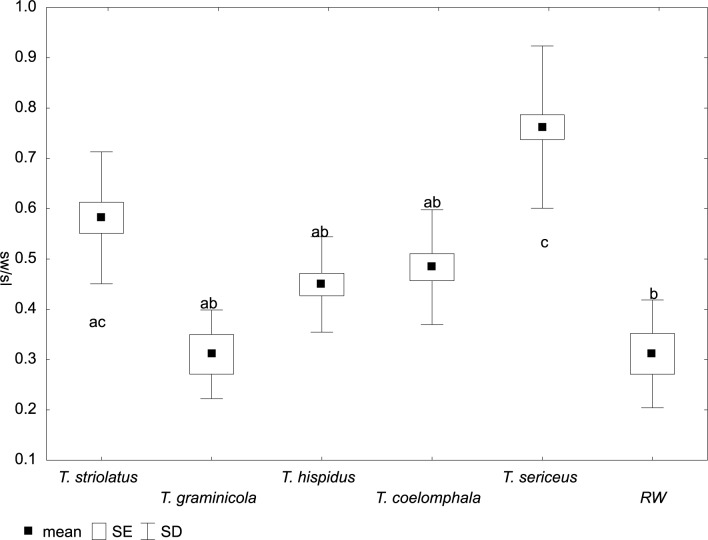
Variation of bursa copulatrix width/length ratio (sw/sl) in *Trochulus* taxa and Ruine Waldau population (RW). Letters a–c indicate significant differences determined by the Kruskal–Wallis test (*p* < 0.05).

**Fig 9:**
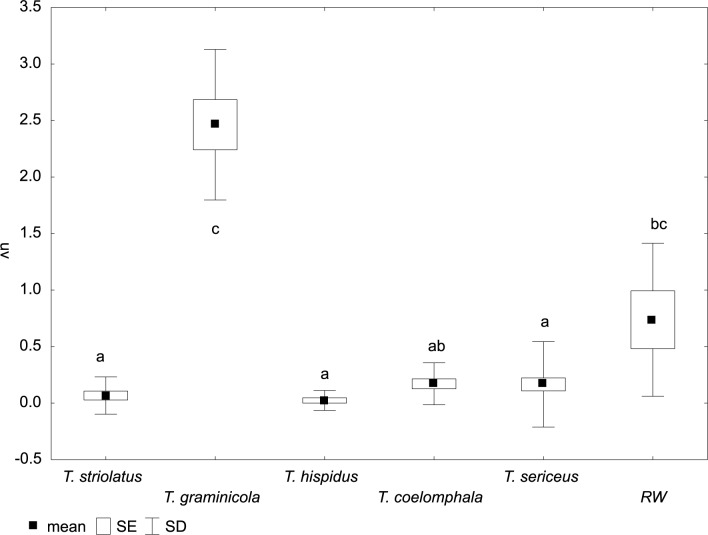
Variation of length of upper vagina (uv) in *Trochulus* taxa and Ruine Waldau population (RW). Letters a–c indicate significant differences determined by the Kruskal–Wallis test (*p* < 0.05).

In the CDA of genital measurements, the first discriminant function (Can1) accounted for 57.1% of the variance and the second (Can2) accounted for 22.4% (Fig 7). The highest loadings (Table 4) on the first function were for bursa copulatrix width/length ratio (sw/sl) and for the upper vagina (uv). The first function clearly separated *T*. *graminicola* specimens that were characterized by smaller values of sw/sl and longer uv (range 0.77–3.00 mm) (Figs 8 and 9). The first trait (sw/sl) was statistically significant between *T*. *sericeus* and all other groups except for *T*. *striolatus* (Fig 8). This means that *T*. *sericeus* has the most round bursa copulatrix (sw/sl = 0.46–1), whereas *T*. *graminicola* and RW specimens the most elongated (sw/sl = 0.22–0.44 and 0.17–0.46, respectively) among all examined taxa. The RW specimens, however, could not be distinguished from *T*. *hispidus* and *T*. *coelomphala*. The highest loadings on the second function (Table 4) were ascribed to the flagellum/epiphallus ratio (fl/ep) and length of the epiphallus (ep). These features appeared to be very variable within the species/population and could not reliably separate them. For example, all specimens of *T*. *striolatus* included in the analysis intermixed either with *T*. *hispidus*, *T*. *coelomphala* or *T*. *sericeus*. Similarly, the RW specimens, *T*. *hispidus* and *T*. *coelomphala* overlapped with each other to too great an extent (Fig 7).

**Table 4:** Canonical coefficients of discriminant analysis performed on genital measurements.

Variable	Standardized canonical discriminant function coefficients	
	Can1	Can2
sw/sl	**-0.843**	0.233
sd/sl	0.292	-0.476
uv	**0.808**	-0.566
sl	-0.045	0.958
ep/p	0.626	-0.509
is/os	-0.533	-0.175
sw	-0.599	-0.663
fl	-0.468	1.030
fl/ep	0.345	**-1.539**
ep	-0.203	**-1.266**
p	0.399	-0.082
Eigenvalue	4.318	1.689
Cum. Prop. (%)	57.1	79.5

The highest values are in **bold**. For abbreviations, see Materials and methods.

The CDA correctly classified 85.7% of *T*. *hispidus*, 97.7% of *T*. *sericeus*, 72.2% of *T*. *striolatus*, 86.3% of *T*. *coelomphala*, 88.9% of *T*. *graminicola* and 85.7% of RW specimens. The overall classification accuracy was 88.1%.

### Mitochondrial gene tree

**Fig 10:**
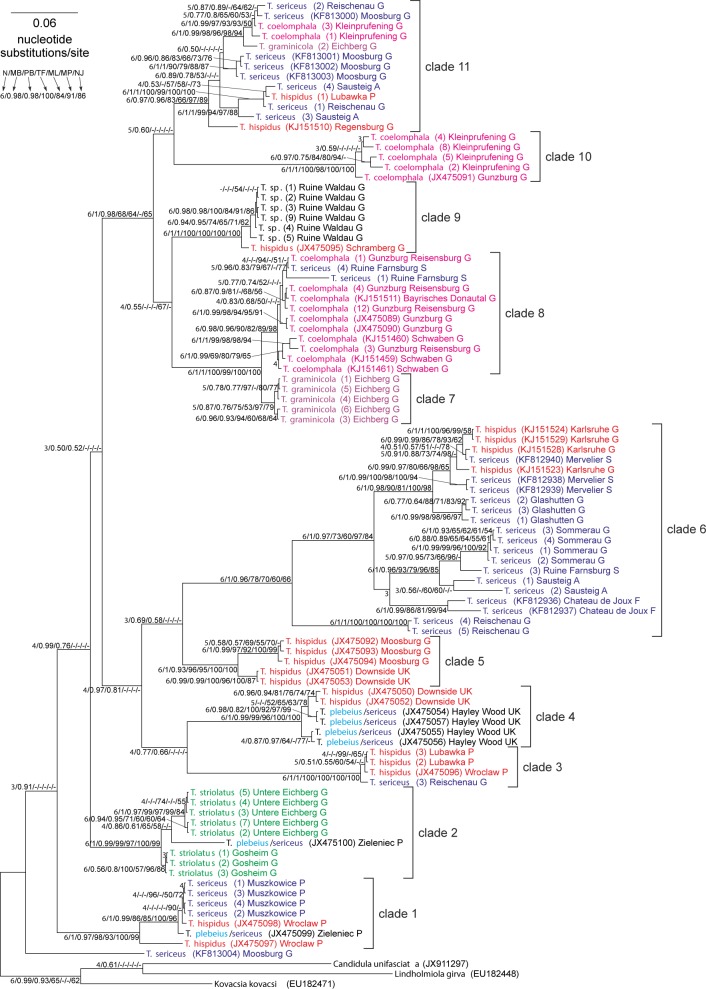
MrBayes tree of *Trochulus* COI gene sequences for the long dataset. Numbers at nodes, in the order shown, correspond to: the number of approaches/software (from six possible) supporting a given node (**N**), posterior probabilities estimated in MrBayes (MB) and PhyloBayes (PB) as well as bootstrap support values obtained in TreeFinder (TF) and in PAUP by maximal likelihood (ML), maximum parsimony (MP) and neighbour-joining (NJ) methods. Values of the posterior probabilities and bootstrap percentages lower than 0.50 and 50%, respectively, were omitted or indicated by a dash “-”.

**Fig 11:**
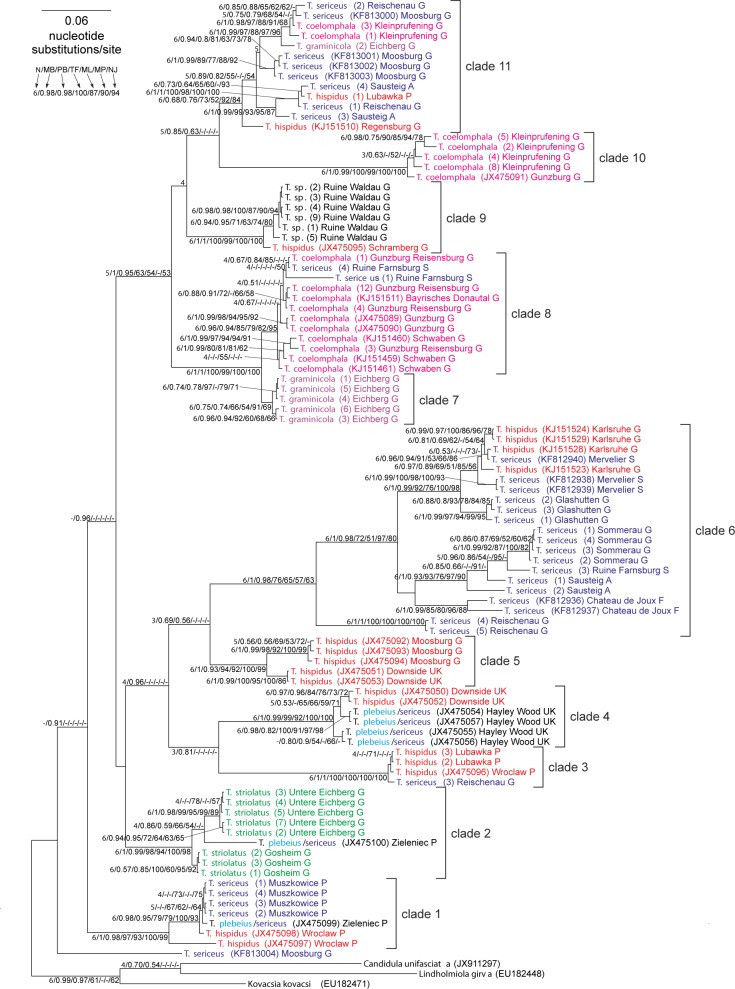
MrBayes tree of *Trochulus* COI gene sequences for the short dataset. For other explanations see Fig 10.

Phylogenetic analyses based on different methods and two sets (long and short) produced very similar tree topologies (Figs 10 and 11). The sister position to the remaining *Trochulus* taxa is occupied by *T*. *sericeus* from Moosburg (Germany) but it is not strongly supported. In the trees, eleven main clades usually with high and very high support values could be recognized. Ten of them obtained posterior probability larger than 0.96 or bootstrap percentage greater than 96% by at least one of applied methods for the long dataset. However, none of the morphology based taxa (or morphospecies) was monophyletic in the tree. Their representatives were located in different clades. Considering deep relationships between the clades, only clades 7–11 were grouped with quite significant support. It should also be noted that members of *T*. *hispidus* complex, i.e. *T*. *hispidus* and *T*. *sericeus* distributed in clades 3, 4, 5 and 6, clustered together forming one major clade inferred by four of six applied methods/software on two datasets and strongly supported by the MrBayes approach.

Interestingly, there were several examples where sequences from a single locality of the same species were distant from each other in the trees. *Trochulus sericeus* from Moosburg (Germany) was located at the base of the tree and in clade 11; *T*. *sericeus* from Reischenau (Germany) was in clades 3, 6 and 11; *T*. *sericeus* from Sausteig (Austria) was in clades 6 and 11; *T*. *hispidus* from Wrocław (Poland) was in clades 1 and 3; *T*. *hispidus* from Lubawka (Poland) was in clades 3 and 11; and *T*. *coelomphala* from Kleinprüfening (Germany) was in clades 10 and 11.

Although clade 2 comprised all eight studied *T*. *striolatus* sequences from Gosheim and Untere Eichberg (Germany), it also included a sequence ascribed to *T*. *plebeius*/*sericeus*. The *T*. *striolatus* sequences showed low genetic divergence. Their intragroup *p*-distance expressed as a percentage was 1.21% ± 0.31% and their *p*-distance to the remaining *Trochulus* sequences was 14.32% ± 0.96%. On the other hand, clade 5 contained only *T*. *hispidus* whereas clade 10 contained only *T*. *coelomphala*, but these species were also widely distributed in other clades. Additionally, clade 6 consisted of only *T*. *sericeus* because specimens annotated in GenBank as *T*. *hispidus* complex were morphologically attributed to *T*. *sericeus* by Duda et al. [27] and Kruckenhauser et al. [16].

Similarly, clade 7 grouped five sequences obtained from *T*. *graminicola* collected in Eichberg (Germany) but one sequence of this species was also significantly located in clade 11. The five sequences of *T*. *graminicola* showed very low within-group genetic divergence. Their *p*-distance was 0.34% ± 0.16%, more than 40 times less than the *p*-distance of these sequences to the remaining *Trochulus* sequences (13.79% ± 0.92%). The sixth *T*. *graminicola* sequence was remarkably diverged from the other five sequences (12.52% ± 1.26%) and clustered significantly with two sequences of *T*. *coelomphala* from Kleinprüfening (Germany) and two of *T*. *sericeus* from Moosburg and Reischenau (Germany). Our results indicate that the five *T*. *graminicola* sequences (in clade 7) are the most closely related to clade 8, including many *T*. *coelomphala* samples from different localities in Germany and two *T*. *sericeus* specimens from Ruine Farnsburg (Switzerland).

All specimens from RW formed a monophyletic well-supported clade, which was joined with very high support with *T*. *hispidus* from the closely located Schramberg in Germany (6.2 km away), forming clade 9. The position of this clade was the only difference between the tree topologies for the two datasets. In the tree for the long set (Fig 10), this clade clustered with clades 7 and 8, whereas for the short dataset (Fig 11), it was sister for clades 10 and 11. Such positions were obtained by four of six approaches/software in each of these datasets. The intragroup *p*-distance between snails from RW was very low, 0.31% ± 0.13%, whereas the *p*-distance to the remaining *Trochulus* taxa was very large: 13.89% ± 0.94%.

### Molecular delimitation of species boundaries

**Fig 12:**
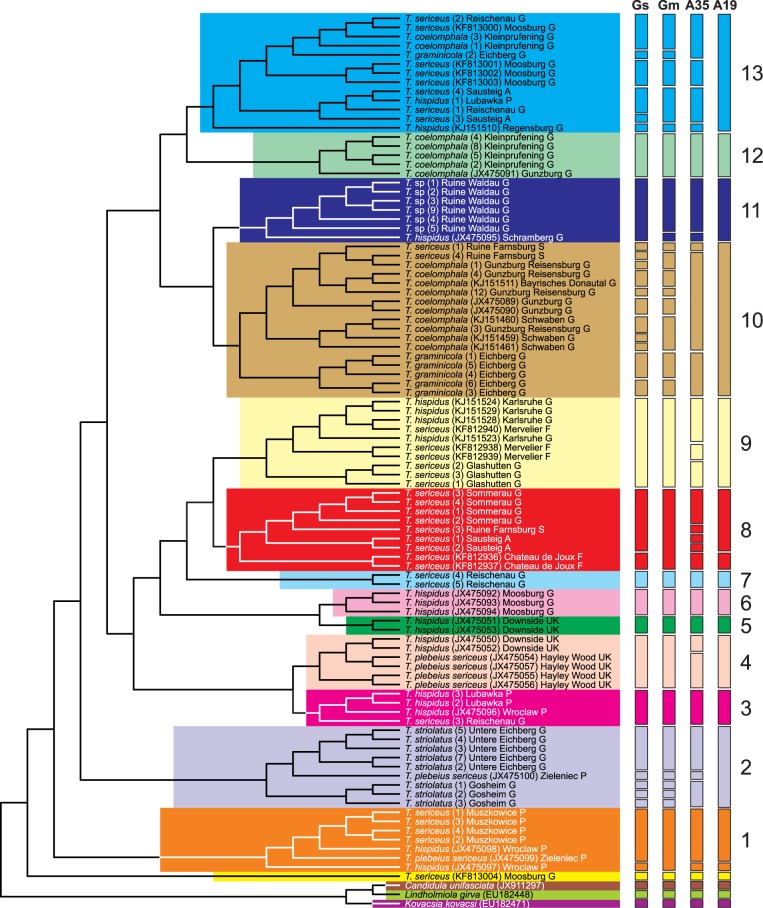
Clusters of delimited species based on the ultrametric MrBayes tree for the long dataset obtained with GMYC single- (Gs) and multiple-threshold (Gm) models as well as two ABGD that recognized the largest and the smallest number of potential species (A35 and A19, respectively). The most numerous clusters found by at least one method were marked by individual colours.

We applied two delimitation methods, ABGD and GMYC to determine species boundaries based on the long alignment of COI sequences (Fig 12). Both single- and multiple-threshold GMYC models fitted the data significantly better (Likelihood Ratio test, *p*-values 0.0007 and 4.6·10^−5^, respectively) than the null model assuming that the entire sample derives from a single species with uniform branching, in contrast to the alternative hypothesis about several independently evolving species lineages. The number of all delimited species including singletons (species represented by a single sample) was for the single GMYC method 38 (20 clusters + 18 singletons) and for the multiple GMYC method 33 (20 clusters + 13 singletons). The results obtained with the ABGD method were similar but dependent on a priori threshold value. In total, eight partition schemes were proposed. The largest number of 35 species (22 clusters + 13 singletons) was identified for the prior maximal distance *p* from 0.001 to 0.002783, whereas the smallest number 19 (14 clusters + 5 singletons) for *p* = 0.035938. These two extremes were presented in Fig 12 for the ABGD method.

In Fig 12, we have marked by individual colours the most numerous species groups found by at least one method. There are 17 such groups consisting of 13 clusters (with at least 2 species) and 4 singletons. The clear singletons are three snails that were used as outgroup and *T*. *sericeus* (KF813004) from Moosburg, which is sister to other *Trochulus*. Almost all 13 clusters correspond to very well-supported clades or lineages in the phylogenetic tree (Fig 10). Five of these clusters (5–8, 12) comprise sequences with the same species assignment. However, in cluster 8, three methods propose separation of some samples as separate species. Similarly, within clusters 2 and 10, samples assigned to the same species and grouped together in the tree are considered as separate species by some methods (usually GMYC). In clusters 2 and 3, one sequence has different taxonomic name than others: *T*. *plebeius/sericeus* (JX475100) and *T*. *sericeus* (3). The former is considered as a separate species from *T*. *striolatus* by some methods but the latter is always grouped to one cluster with *T*. *hispidus*. There are many clusters that contain a mixture of samples with various species names. Even if we take into account methods that recognized many potential species, there are still subclusters within cluster 13 that include sequences with different species affiliations.

### Microsatellite clustering

**Fig 13:**
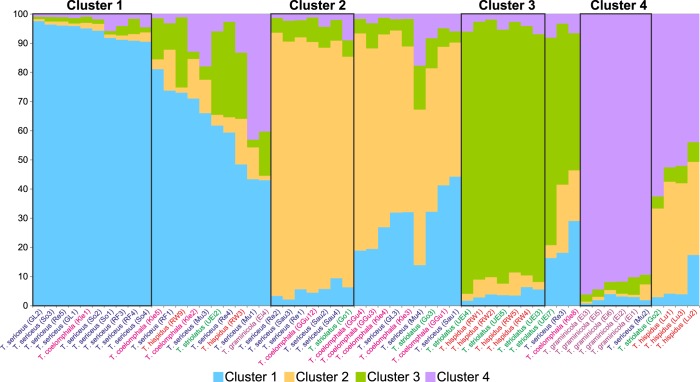
Genetic structure of *Trochulus* according to microsatellite markers. Each individual is ascribed bars whose colour denotes one of four genetic clusters, whereas length corresponds to a probability of its assignment to the cluster. Cases classified to these clusters according to the appropriate probability are outlined by a rectangle.

Both the Delta K method and L(K) plot indicated K = 4 as the most probable number of distinct genetic clusters for microsatellites (data not shown). Thirty individuals were attributed to one of these four clusters with posterior probability larger than 0.9 while 26 individuals had mixed affiliation to two (20 individuals), three (four individuals) or four (two individuals) clusters with a probability of belonging to the clusters between 0.1 and 0.9 (Fig 13). There was no cluster that would contain representatives of only one *Trochulus* species. Clusters 1 and 2 were dominated by *T*. *sericeus* but also grouped one *T*. *coelomphala* individual in each of these clusters and *T*. *sericeus* also in cluster 2. Cluster 3 comprised four *T*. *hispidus* and three *T*. *striolatus* representatives. Five of six *T*. *graminicola* snails were grouped into cluster 4, which also included one *T*. *sericeus*. Many other individuals of all studied species were ascribed to mixed clusters.

### Testing consistency of different approaches

The consistency of an individual’s classification by morphology described by metric features, geographic location and genetic analyses was checked by G-test of independence based on appropriate contingency tables. The analyses showed significant (with *p* < 0.05) relationships between all studied classifications with the exception of microsatellite data when 12 microsatellite groups were considered (Table 5). However, the relationships of microsatellite data with others were significant, when four clusters and one mixed group were assumed.

**Table 5:** Corrected *p*-values given by G-test for contingency tables classifying individuals according to species, geographic location, morphology, clade based on mitochondrial COI phylogeny and microsatellite clusters.

	Species	Location	Morphology	COI	Microsatellites
Species	-	1.8E-08	0.0000	2.2E-09	0.1188
Location	1.8E-08	-	1.4E-08	0.0168	1.0000
Morphology	0.0000	1.4E-08	-	3.4E-09	0.1265
COI	2.2E-09	0.0112	3.4E-09	-	1.0000
Microsatellites	4.7E-05	0.0123	4.7E-05	0.0023	-

The upper triangle refers to the analysis in which 12 microsatellite groups (four clear clusters and eight mixed assignments of individuals) were considered, whereas the lower triangle refers to studies with five microsatellite groups (four clear clusters and one including mixed groupings).

We also observed many significant correlations in the Mantel test between distance matrices expressing differences in spatial distribution (geographical and altitude location), shell metric morphology, COI sequences and microsatellites of studied snail samples (Table 6). However, correlation coefficients were not high. Disregarding the relationship between geographic and altitude distance, correlation coefficients of about 0.2 were found for differences in COI sequences with distances in geographic location, altitude and shell morphology as well as between microsatellite and shell morphology distances. The two genetic distances based on mtDNA and microsatellites were weakly correlated (*r* = 0.137). Shell morphology also revealed weak correlation with altitude distance and a marginal correlation with geographic distance. No significant correlation was observed for microsatellites with geographical and altitude distances. We did not find a significant relationship between differences in genitalia measurements and geographic distance (*r* = 0.007, *p* = 0.420), but there was a weak correlation with altitude distance (*r* = 0.105, *p* = 0.018).

**Table 6:** Correlation coefficient (upper triangle) and corrected *p*-values (lower triangle) resulting from a Mantel test comparing matrices for: geographical and altitude distance between sites and where snails were found, as well as distances in shell morphology, COI sequences and microsatellites.

	Geographic distance	Altitude distance	Shell morphology	COI distance	Microsatellites distance
Geographic distance	-	0.405	0.060	0.237	-0.084
Altitude distance	0.0002	-	0.122	0.204	-0.042
Shell morphology	0.0056	0.0002	-	0.210	0.215
COI distance	0.0002	0.0002	0.0002	-	0.137
Microsatellites distance	0.9059	0.9059	0.0027	0.001	-

## Discussion

### Taxonomic position of *T*. *graminicola* and Ruine Waldau snails

The multivariate analysis of morphological variation in *Trochulus* taxa revealed segregation of some studied specimens into three separate species as recognized by Kerney et al. [37]. There were clear boundaries separating *T*. *graminicola*, *T*. *striolatus* and *T*. *sericeus* based on shell traits such as the umbilicus diameter and relative umbilicus diameter (Fig 3). However, the morphometric limits of *T*. *graminicola* were less evident with comparison to *T*. *hispidus* and *T*. *coelomphala* as well as to the RW specimens. Conversely, *T*. *graminicola* could be clearly distinguished from the remaining taxa based on the reproductive system, in particular long upper vagina (Fig 9) and to a lesser extent slender bursa copulatrix (Fig 8). In agreement with that, our genetic data also showed the distinctness of almost all *T*. *graminicola* COI sequences, which formed the significantly supported clade. This clade was considered as a separate species by five of eight ABGD partition schemes. GMYC methods recognized two clusters in this clade. These specimens exhibited very low intragroup sequence divergence (0.34%) and a very large difference from the remaining *Trochulus* taxa (13.8%). This may imply their common and recent evolutionary history associated with allopatric fragmentation, which finally led to speciation. The genetic *p*-distance, 13.8% between *T*. *graminicola* and the remaining *Trochulus* sequences, was in the range 9.5% to 20.9% found between morphologically well-distinguished *Trochulus* species, i.e. *T*. *striolatus* and *T*. *villosulus* as well as *T*. *striolatus* and *T*. *lubomirskii* [16], respectively. In agreement with that, five of six *T*. *graminicola* individuals were grouped together in microsatellite cluster 4, which also included one *T*. *sericeus*. One case of the second species in this cluster may be caused by independent evolution of the same allele due to the high mutation rate of microsatellites. The sixth *T*. *graminicola* individual showed mixed affiliation to clusters 1, 3 and 4.

**Fig 14:**
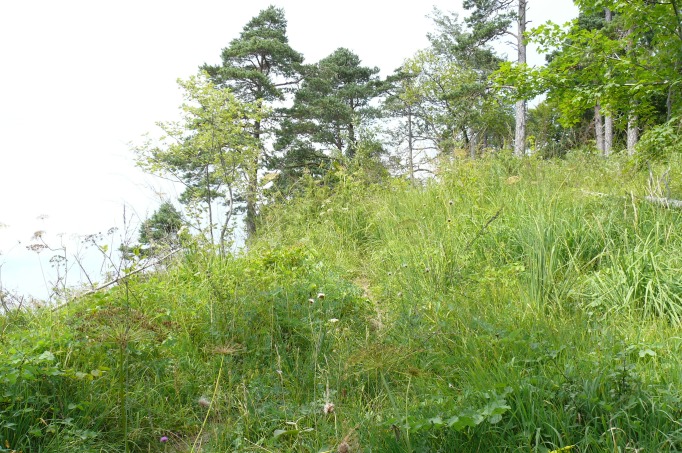
Calamagrostio variae-Pinetum association in Mt. Eichberg, Achdorf near Blumberg, Germany—habitat of *T*. *graminicola*.

The current distribution area of *T*. *graminicola* remained ice-free during the Last Glacial Maximum [69], thus the species could have survived at least the last glaciation within the southern edge of Südschwarzwald. Moreover, if its limited occurrence to a single mountain is confirmed, it will suggest that *T*. *graminicola* evolved in this region and never dispersed. This may result from its strict ecological requirements. This stenoecious species inhabits a narrow ecological niche restricted exclusively to the patches of Calamagrostio variae-Pinetum association (Fig 14), a pioneer or permanent community that occurs on steep marl slopes, with shallow, lime-rich, humus-poor clay soils. This community is associated with a warmer, Atlantic-influenced climate and known from southern Europe (Randalpen, Alpenvorlandes and Schwäbischen Alb) [70]. In southern Germany it is scarce and exists only in small stands. The site of *T*. *graminicola* is situated in the Schwarzwald-Baar region which directly borders the Schwäbischen Alb. The habitat from which we collected the snails represents the typical community but the pine trees are currently rather sparse. This is connected with the fact that the community can be a stage of succession between rocky swards and the forest [41,70]. The locality is thus well illuminated and heated, as is confirmed by the presence of many thermophilous and photophilous plants (e.g. *Laserpitium latifolium*, *Cytisus nigricans*, *Anthericum ramosum*, *Teucrium chamaedrys*, *Thesium bavarum*, *Coronilla coronata*). We found all of the plant species listed in Falkner [32] except for Norway spruce (*Picea abies*), European beech (*Fagus sylvatica*) (both probably harvested earlier) and *Gentiana germanica* s.l. (that is visible only in spring). This plant community is very rare [70]; as it also forms the habitat and only known location for this restricted endemic snail, it is clearly worthy of protection.

As shown by the very restricted ranges and habitat specialization of *T*. *piccardi* [71], *T*. *montanus*, *T*. *biconicus*, *T*. *caelatus* [38] and *T*. *oreinos* [26,72], regional speciation with local persistence seems to be common within the genus *Trochulus* [13,16,27,71]. In general, the poor dispersal power of snails give rise to rather clear patterns of genetic differentiation related to distance; gene flow is limited [73]. Here, however, one *T*. *graminicola* sample related more closely to other *Trochulus* species, which may suggest a presence of gene flow between these snails. It demonstrates that the observed differences between reproductive systems may not be sufficient to isolate reproductively the *Trochulus* species. Our data could also suggest the early stages of divergence of genital traits in *T*. *graminicola* that may have evolved with locally restricted but ongoing gene flow. Therefore, its ecological requirements and consequently limited geographical distribution could contribute to the evolution of reproductive isolation.

The specimens from RW were tentatively assigned to *T*. *graminicola* on the basis of shell morphology and their occurrence at a small distance (ca. 34 km in a straight line) from the type locality [33]. Our integrative studies of these snails could not, however, confirm this view. In the CDA analyses, based on both shell and genital measurements, the RW specimens were distributed among *T*. *hispidus* and *T*. *coelomphala* and, thus, separated from *T*. *graminicola*. In the phylogenetic tree (Fig 10), the RW specimens formed the significant monophyletic mtDNA group, which clustered with *T*. *hispidus* from Schramberg (Germany) but did not join directly with *T*. *graminicola*. The multiple-threshold GMYC method and three ABGD partition schemes recognized the RW specimens as one species, whereas the single-threshold GMYC and five ABGD partition schemes added to this cluster *T*. *hispidus* from Schramberg (Fig 12). In the tree based on the long alignment dataset (Fig 10), the clade was sister to the clade including *T*. *graminicola*, *T*. *coelomphala* and *T*. *sericeus*. The RW sample showed high sequence divergence (mean *p*-distance 12.18% ± 1.19%) from the *T*. *graminicola* clade. Additionally, the genetic cluster of the RW specimens, determined in the microsatellite analysis, also supported their separation from *T*. *graminicola* (Fig 13). They also inhabit different biotopes, despite similar elevations (Table 1). *Trochulus* from RW, unlike *T*. *graminicola*, originates from a castle ruin, situated close to human settlements, a typical habitat for *T*. *hispidus* and *T*. *striolatus*. In light of all the evidence, the suggestion that *Trochulus* from RW is conspecific with *T*. *graminicola* [33] is not justified. It formed a monophyletic clade, with high genetic distances to other clades (13.9%). This is, however, contrary to the results of microsatellite analysis that suggested a high gene flow between the RW population and many other species (Fig 13). Considering *T*. *hispidus* as a paraphyletic species complex and general high mitochondrial divergence in *Trochulus* species [16,28,29,31], as well as morphological similarity of the RW specimens to both *T*. *hispidus* and *T*. *coelomphala* and their low classification success of 43% and 86% in shell and genitalia, respectively, it seems that the taxonomic status of RW specimens must remain unresolved. Temporarily, it can be included in the *T*. *hispidus* complex.

### Distribution of morphospecies in the phylogenetic tree

Species recognised on morphological criteria (morphospecies) did not correspond to monophyletic clades identified by molecular data but were scattered over the phylogenetic tree. The species delimitation methods used identified at least 14 potential species. Of these, five clades in the phylogenetic tree contained two morphospecies (clades 1, 2, 3, 4 and 8) and clade 11 consisted of four mixed taxa: *T*. *hispidus*, *T*. *sericeus*, *T*. *graminicola* and *T*. *coelomphala*. The significant grouping of sequences from different species suggests some gene flow, hybridization or incomplete lineage sorting between them. Alternatively, they may represent a smaller number of species or populations showing great morphological variation related to environment. Gene exchange could happen between different species inhabiting the same region, i.e. between: *T*. *hispidus* and *T*. *sericeus* in Lower Silesia (clade 1) and *T*. *hispidus* and *T*. *plebeius/sericeus* in southern Great Britain (clade 4), as well as *T*. *sericeus* with *T*. *hispidus*, *T*. *graminicola* and *T*. *coelomphala* in Germany (clade 11). Similarly, hybridization could have occurred between *T*. *coelomphala* and *T*. *sericeus* snails in neighbouring regions in Germany and Switzerland (clade 8). Interestingly, gene flow over longer distances seems indicated by similarities between German *T*. *striolatus* and Polish *T*. *plebeius/sericeus* (clade 2), German *T*. *sericeus* and Polish *T*. *hispidus* (clade 3) and Austrian *T*. *sericeus* and Polish *T*. *hispidus* (clade 11). In clades 1, 3 and 11, no species delimitation method, recognized all morphospecies as separate clusters. In clade 4, most of these methods also considered different morphospieces as one cluster, which suggests that these morphospecies should not be considered as separate species.

*Trochulus hispidus* collected from distant locations in Downside (Great Britain) and Moosburg (Germany) were significantly clustered in clade 5 with exclusion of other English and German *T*. *hispidus*. This could also indicate some gene flow between the English and continental populations or a common ancestry because all species delimitation methods consider the Downside and Moosburg populations as a separate species.

### Integrating morphological and molecular analyses of other taxa

Most of the individuals of *T*. *coelomphala* placed in the ordination area overlapped *T*. *hispidus* in both shell and genital analyses (Figs 3 and 7). Likewise, this evidence was confirmed using the CDA method. Based on this analysis, *a posteriori* classification of shell morphology indicated that only 69% of *a priori*-identified specimens of *T*. *coelomphala* were correctly classified (Table 2). There was no perfect classification success rate in genital morphology (86%). It appears that the interspecific boundary was disturbed either due to high morphological variation and/or scarce taxon sampling in the present work. In our phylogram (Fig 10), all specimens tentatively determined as *T*. *coelomphala* based on their geographic origin (the Bavarian Danube valley [36]) were separated into three clades, with only one being monophyletic (clade 10) and consisting of five snails coming from two localities examined in this study. All these specimens were recognized as one species by all methods. The remaining two clades (8 and 11) also contained other *Trochulus* morphotypes from distant geographic regions such as Baselland (Switzerland), Swabia, Upper Bavaria, Schwarzwald-Baar (Germany), Bregenz Forest (Austria) and Central Sudetes (Poland). The clustering of *T*. *coelomphala* with *T*. *sericeus* in these clades would suggest gene flow between these snails or incomplete mitochondrial lineage sorting, if we assume that they are separate species. However, no delimitation method considered them as separate species in clade 11 and in clade 8. Most methods grouped *T*. *coelomphala* with at least one *T*. *sericeus* into one cluster. The analysis of microsatellite markers revealed a low degree of genetic differentiation among the examined *T*. *coelomphala* populations, and no cluster contained exclusively *T*. *coelomphala* individuals. Each snail was classified into two clusters, 1 and 2, six were assigned simultaneously to clusters 1 and 2, and one individual showed mixed affiliation to clusters 1 and 3, whereas the other was grouped to clusters 1, 2 and 3. Both here and in the results obtained by other authors [27], the taxonomic status of *T*. *coelomphala* remains unclear.

Individuals morphologically resembling *T*. *hispidus* and *T*. *sericeus* should be considered as *T*. *hispidus* complex [27] because none of them meets the criterion of monophyly [16]. In these two parallel studies, nine highly differentiated mitochondrial clades were identified, also revealing no morphometric differentiation between the clades. Species delimitation methods also clustered *T*. *hispidus* and *T*. *sericeus* together. We identified three main *T*. *hispidus* complex clades in the phylogenetic tree (1, 3+4+5 and 11). Clades 1, 3, 4 and 5 consisted exclusively of both morphotypes. Clade 1 was clearly separated from clades 3, 4 and 5, which grouped together and formed one big clade. The third separated clade (11) besides *T*. *hispidus* and *T*. *sericeus* also contained two *T*. *coelomphala* sequences and one *T*. *graminicola* sequence, suggesting some gene flow to them from members of the *T*. *hispidus* complex or incomplete lineage sorting of mtDNA. At least five cases of hybridization between *T*. *hispidus* and *T*. *sericeus* can be assumed. Two of them concern geographically distant populations in Poland and Germany/Austria. Interesting instances are *T*. *hispidus* from Downside in Great Britain and Moosburg an der Isar in Germany that form the strongly supported clade 5. This could indicate that the populations from the British Isles and continental Europe maintained genetic similarity, e.g. during and shortly after the last glacial period when the sea level was low and these two regions were not separated by the English Channel but connected by the Weald–Artois anticline and Doggerland, which was flooded by rising sea around 6,500–6,200 BC [74]. Nevertheless, the genetic differences between these populations are so large that all species delimitation methods considered them as separate species.

Interestingly, clade 6 contained individuals with a small relative diameter, thus belonging to *T*. *sericeus* [28] and corresponding to clade 8 in trees of other authors [16,27]. In the study by Duda et al. [27], clade 8 containing exclusively *T*. *sericeus* was also the only one in which both morphological and genetic data were consistent. In our trees, four snails from Karlsruhe, i.e. those of clade 8 [27], were nested within our clade 6. Species delimitation methods recognized at least three potential species within clade 6, but did not separate *T*. *hispidus* and *T*. *sericeus*.

In contrast to that, *T*. *hispidus* and *T*. *sericeus* were never grouped together into well-defined microsatellite clusters, which may result from smaller sampling of microsatellite than mitochondrial data. Individuals of *T*. *sericeus* dominated in two microsatellite clusters (1 and 2); in addition, one each of *T*. *coelomphala* and *T*. *striolatus* snails were included in cluster 2. The presence of these single members of other species in these clusters may result from the high degree of microsatellite mutation and development of non-homologous alleles of the same size by chance.

Studies on shell morphology showed only a limited overlap between *T*. *hispidus* and *T*. *sericeus* morphotypes (Fig 3). The major umbilicus diameter (U) and relative umbilicus diameter (U/D) best described the differences between these two ecophenotypes (Figs 5 and 6). However, this morphometric separation does not agree with the mtDNA trees, in which these taxa are grouped together in many clades. This is because these two different morphospecies, *T*. *hispidus* and *T*. *plebeius/sericeus*, from the same geographical region, are closely related [28], and can interbreed giving fertile offspring [75]. Moreover, under constant laboratory conditions the average shell shape of *T*. *hispidus* changed significantly from flat with a wide umbilicus to more globular or even elevated with a narrower umbilicus, thus resembling *T*. *sericeus*. Therefore, this phenotypic plasticity could explain the evolution of sympatric polymorphism in this species complex [75]. The absence of significant anatomical differences between these morphotypes recorded in this and other studies [27,28] provides additional evidence for the lack of reproductive barriers as indicated by genetic studies.

The coexistence of distinct gene pools in sympatry strongly indicates the presence of separate species. In allopatry, however, it is harder to assess the significance of observed differences in terms of the conventional biological species concept. While the efficiency of microsatellites in data to identify species has been recently demonstrated for various groups of plants and animals [1,76–80], our studies did not show the clear separation of morphological species into microsatellite clusters. Almost half of the snails studied showed a mixed classification.

Considering the phylogenetic species concept, there are three possible explanations of the discrepancy between genetic and morphological data regarding the recognition of the two morphospecies *T*. *hispidus* and *T*. *sericeus*: (1) the morphospecies may have diverged recently and a complete barrier to gene flow between them has not yet been established; (2) they belong to the same species but display substantial morphological variation; (3) they were represented by too few individuals (i.e. insufficient sampling), which prevented the identification of separate gene pools. Our results illustrate difficulties in assessing species status as well as problems associated with underlying definitions of species concepts in snails.

The differentiation of *T*. *striolatus* from the *T*. *sericeus* and *T*. *graminicola* examined in this study was clear based on U/D, being intermediate (0.14–0.22) between the two latter taxa (0.05–0.18 and 0.21–0.29, respectively). Despite our limited sampling of *T*. *striolatus* (*n* = 42) these values stayed in accordance with those calculated based on extensive material [25,29]. Therefore, this trait, besides a specific riffle pattern on the shell surface [27], may be useful to discriminate *T*. *striolatus*. Other shell and genital morphological features separated *T*. *striolatus* only to some extent (Figs 5, 8 and 9) because of its extremely high intraspecific shell size differences [27,29], which may be consequences of its ecophenotypic variation. For example, our recent study revealed correlations between the morphological variation of *T*. *striolatus* and altitude as well as other climate variables, which might be explained by this plasticity. As a result, we found no sufficient justification for its infraspecific classification (Proćków et al. unpublished work). Admittedly, a slight morphological differentiation in the cross section of the penis between subspecies (*T*. *striolatus striolatus*, *T*. *s*. *danubialis* and *T*. *s*. *juvavensis*) was detected but it was based on a limited sampling dataset (*n* = 26 [27]). In agreement with that, the three different subspecies were not clearly separated on the basis of the COI mtDNA data [16]. On the other hand, *T*. *striolatus* appears to be a good species based on morphology and COI, and its monophyly was also evidenced in this and previous studies [16,29]. Clade 2 in mtDNA trees was the only one that included all the studied snails of one species, namely *T*. *striolatus*, although it also contained one sequence ascribed to *T*. *plebeius/sericeus* from Zieleniec (Poland), suggesting gene flow between these taxa or incomplete lineage sorting. However, not all species delimitation methods recognized all *T*. *striolatus* samples as one species. *Trochulus striolatus* did not form pure microsatellite clusters either but mixed with other species. This may result from incomplete sorting of microsatellite alleles into these lineages or independent evolution of the same microsatellite alleles in these species. Patterns of divergence with gene flow were observed in both invertebrate [81,82] and vertebrate species [83–85].

## Conclusions

General comparisons of results for shell and genitalia metrics and spatial distribution as well as genetic analyses revealed that these features are not completely independent and show some correlations. Altitude showed greater influence on shell and genitalia morphology than geographic distance. Mitochondrial genetic variation revealed a stronger relationship with shell morphology and spatial distribution than microsatellite data. However, correlation coefficients between these features were at best weak although significant. This indicates that geographic and altitude location cannot fully explain shell, genitalia and genetic variation. The same constraint applies to comparisons of shell morphology with genetic variation and to the comparison of mitochondrial with microsatellite data. Most species delimitation methods recognise *T*. *graminicola* as a distinct species, based on its consistent recognition as a separate mitochondrial clade, its domination in one microsatellite cluster and the distinction from other species in genitalia characteristics. *Trochulus sericeus* created a few separate clades in the COI phylogeny, dominating in two microsatellite clusters and differing from other *Trochulus* in shell morphology. *Trochulus striolatus* was grouped into one clear mitochondrial clade and showed different shell morphology in comparison to other species. However, both mitochondrial and microsatellite data indicated that there is no clear separation of all *Trochulus* species; samples of the different morphospecies studied were mixed, suggesting continuing gene flow between them. Alternatively, they are descendants of an ancestral widespread population, from which the recently separated populations inherited the same markers and differentiated morphologically due to local environment. Additional research is required to understand the evolutionary history of *Trochulus* well. Our studies constitute an important step towards the comprehensive revision of the genus, in which taxonomic status of many species remains poorly understood.

## Supporting Information

S1 Dataset Shell and genitalia measurements of all specimens examined. (XLSX)

## References

[1] Duminil J, Kenfack D, Viscosi V, Grumiau L, Hardy OJ. Testing species delimitation in sympatric species complexes: The case of an African tropical tree, *Carapa* spp. (Meliaceae). Mol Phylogenet Evol. 2012;62: 275–285. 10.1016/j.ympev.2011.09.020 22019936

[2] de Queiroz K. Different species problems and their resolution. BioEssays. 2005;27: 1263–1269. 10.1002/bies.20325 16299765

[3] Sites JW, Marshall JC. Delimiting species: A Renaissance issue in systematic biology. Trends Ecol Evol. 2003;18: 462–470.

[4] Hebert PDN, Ratnasingham S, deWaard JR. Barcoding animal life: cytochrome c oxidase subunit 1 divergences among closely related species. Proc Biol Sci. 2003;270 Suppl: S96–9. 10.1098/rsbl.2003.0025 12952648 PMC1698023

[5] Kekkonen M, Hebert PDN. DNA barcode-based delineation of putative species: Efficient start for taxonomic workflows. Mol Ecol Resour. 2014;14: 706–715. 10.1111/1755-0998.12233 24479435 PMC4264940

[6] Purty R, Chatterjee S. DNA Barcoding: An Effective Technique in Molecular Taxonomy. Austin J Biotechnol Bioeng. 2016;3: 1059.

[7] Selkoe KA, Toonen RJ. Microsatellites for ecologists: A practical guide to using and evaluating microsatellite markers. Ecology Letters. 2006 pp. 615–629. 10.1111/j.1461-0248.2006.00889.x 16643306

[8] Hoshino AA, Bravo JP, Nobile PM, Morelli KA. Microsatellites as tools for genetic diversity analysis In: Caliskan M, editor. Genetic diversity in microorganisms. Croatia: INTECH; 2012 pp. 149–170.

[9] Dépraz A, Hausser J, Pfenninger M. A species delimitation approach in the *Trochulus sericeus/hispidus* complex reveals two cryptic species within a sharp contact zone. BMC Evol Biol. 2009;9: 171 10.1186/1471-2148-9-171 19622149 PMC2724411

[10] Arnaud JF, Laval G. Stability of genetic structure and effective population size inferred from temporal changes of microsatellite DNA polymorphisms in the land snail *Helix aspersa* (Gastropoda: Helicidae). Biol J Linn Soc. 2004;82: 89–102.

[11] Schweiger O, Frenzel M, Durka W. Spatial genetic structure in a metapopulation of the land snail *Cepaea nemoralis* (Gastropoda: Helicidae). Mol Ecol. 2004;13: 3645–3655. 10.1111/j.1365-294X.2004.02357.x 15548280

[12] Dépraz A, Cordellier M, Hausser J, Pfenninger M. Postglacial recolonization at a snail’s pace (*Trochulus villosus*): Confronting competing refugia hypotheses using model selection. Mol Ecol. 2008;17: 2449–2462. 10.1111/j.1365-294X.2008.03760.x 18422928

[13] Ursenbacher S, Alvarez C, Armbruster GFJ, Baur B. High population differentiation in the rock-dwelling land snail (*Trochulus caelatus*) endemic to the Swiss Jura Mountains. Conserv Genet. 2010;11: 1265–1271.

[14] Davison A, Blackie RLE, Scothern GP. DNA barcoding of stylommatophoran land snails: A test of existing sequences. Mol Ecol Resour. 2009;9: 1092–1101. 10.1111/j.1755-0998.2009.02559.x 21564847

[15] Sauer J, Hausdorf B. A comparison of DNA-based methods for delimiting species in a Cretan land snail radiation reveals shortcomings of exclusively molecular taxonomy. Cladistics. 2012;28: 300–316. 10.1111/j.1096-0031.2011.00382.x 34872193

[16] Kruckenhauser L, Duda M, Bartel D, Sattmann H, Harl J, Kirchner S, et al Paraphyly and budding speciation in the hairy snail (Pulmonata, Hygromiidae). Zool Scr. 2014;43: 273–288. 10.1111/zsc.12046 25170185 PMC4144147

[17] Carstens BC, Pelletier TA, Reid NM, Satler JD. How to fail at species delimitation. Molecular Ecology. 2013 pp. 4369–4383. 10.1111/mec.12413 23855767

[18] Miralles A, Vences M. New Metrics for Comparison of Taxonomies Reveal Striking Discrepancies among Species Delimitation Methods in Madascincus Lizards. PLoS One. 2013;8. 10.1371/journal.pone.0068242 PMC3710018 23874561

[19] Fontaneto D, Flot JF, Tang CQ. Guidelines for DNA taxonomy, with a focus on the meiofauna. Mar Biodivers. 2015;45: 433–451.

[20] Rannala B. The art and science of species delimitation. Curr Zool. 2015;61: 846–853.

[21] Falkner G. Zur Problematik der Gattung *Trichia* (Pulmonata, Helicidae) in Mitteleuropa. Mitteilungen der Dtsch Malakol Gesellschaft. 1982;3: 30–33.

[22] Locard A. Les coquilles terrestres de France. Ann la Société d’Agriculture, Sci Ind Lyon. 1895;7: 137–248.

[23] Locard A. Les coquilles terrestres de France. Ann la Société d’Agriculture, Sci Ind Lyon. 1896;7: 5–258.

[24] Germain L. Les Helicidae de la faune française. Arch du Muséum d’histoire Nat Lyon. 1929;13: 1–484.

[25] Proćków M. The genus *Trochulus* Chemnitz, 1786 (Gastropoda: Pulmonata: Hygromiidae)–a taxonomic revision. Folia Malacol. 2009;7: 101–176.

[26] Duda M, Sattmann H, Haring E, Bartel D, Winkler H, Harl J, et al Genetic differentiation and shell morphology of *Trochulus oreinos* (Wagner, 1915) and *T*. *hispidus* (Linnaeus, 1758) (Pulmonata: Hygromiidae) in the northeastern Alps. J Molluscan Stud. 2011;77: 30–40. 10.1093/mollus/eyq037 25197157 PMC4153987

[27] Duda M, Kruckenhauser L, Sattmann H, Harl J, Jaksch K, Haring E. Differentiation in the *Trochulus hispidus* complex and related taxa (Pulmonata: Hygromiidae): Morphology, ecology and their relation to phylogeography. J Molluscan Stud. 2014;80: 371–387. 10.1093/mollus/eyu023 25364084 PMC4214462

[28] Proćków M, Mackiewicz P, Pieńkowska JR. Genetic and morphological studies of species status for poorly known endemic *Trochulus phorochaetius* (Bourguignat, 1864) (Gastropoda: Pulmonata: Hygromiidae), and its comparison with closely related taxa. Zool J Linn Soc. 2013;169: 124–143.

[29] Proćków M., Strzała T., Kuźnik-Kowalska E. MP. Morphological similarity and molecular divergence of *Trochulus striolatus* and *T*. *montanus*, and their relationship to sympatric congeners (Gastropoda: Pulmonata: Hygromiidae). Syst Biodivers. 2014;12: 366–384.

[30] Pfenninger M, Schwenk K. Cryptic animal species are homogeneously distributed among taxa and biogeographical regions. BMC Evol Biol. 2007;7: 121 10.1186/1471-2148-7-121 17640383 PMC1939701

[31] Pfenninger M, Hrabakova M, Steinke D, Depraz A. Why do snails have hairs? A Bayesian inference of character evolution. BMC Evol Biol. 2005;5: 59 10.1186/1471-2148-5-59 16271138 PMC1310604

[32] Falkner G. Studien über Trichia Hartmann, I. *Trichia (Trichia) graminicola* n. sp. aus Südbaden (Gastropoda: Helicidae). Arch für Molluskenkd. 1973;103: 209–227.

[33] Horst D. van der. Ein Beitrag zur Kenntnis von *Trichia graminicola* Falkner. Mitteilungen der Zool Gesellschaft Braunau. 1978;3: 125–130.

[34] Welter-Schultes F.W. European non-marine molluscs, a guide for species identification Göttingen: Planet Poster Editions; 2012.

[35] Locard A. Contributions à la faune malacologique française. XII. Études critiques sur les Helix du groupe de l’*Helix rufescens* Pennant (*Helix striolata*, *H*. *rufescens*, *H*. *montana*, *H*. *cælata*, *H*. *circinata*, *H*. *clandestina*). Ann la Société Linnéenne Lyon (Nouvelle Série). 1888;34: 309–370.

[36] Falkner G. Binnenmollusken In: R., Fetcher FG, editor. Weichtiere Europäische Meeres- und Binnenmollusken, Steinbachs Naturführer München: Mosaik Verlag; 1990 pp. 112–280.

[37] Kerney MP, Cameron RAD, Jungbluth JH. Die Landschnecken Nord- und Mitteleuropas. Hamburg und Berlin: Paul Parey; 1983.

[38] Turner H., Kupier J.G.J., Thew N., Bernasconi R., Rüettschi J., Wüthrich M. GM. Atlas der Mollusken der Schweiz und Liechtensteins. Fauna Helvetica 2. Neuchâtel: Centre suisse de cartographie de la faune.; 1998.

[39] Wiktor A. Mięczaki Ziemi Kłodzkiej i gór przyległych. Studium faunistyczno-geograficzne. Poznańskie Tow Przyj Nauk Wydz Mat Przyr Pr Kom Biol. 1964;29: 1–129.

[40] Paul C.R.C. The ecology of mollusca in ancient woodland. J Conchol. 1967;28: 301–327.

[41] Oberdorfer E. Pflanzensoziologische Exkursionsflora. 7. Aufl. Stuttgart: Ulmer; 1994.

[42] Ehrmann P. Mollusken (Weichtiere) In: Brohmer P, Ehrmann P, Ulmer G, editors. Die Tierwelt Mitteleuropas, Vol 2 Leipzig: Quelle and Meyer; 1933 pp. 1–264.

[43] R Developement Core Team. R: A Language and Environment for Statistical Computing. R Found Stat Comput. 2015;1: 409.

[44] Dépraz A, Rathey E, Hausser J. Characterization of 13 polymorphic microsatellite loci for two land snail species, *Trochulus villosus* and *T*. *sericeus* (Gastropoda: Pulmonata: Hygromiidae). Mol Ecol Resour. 2008;8: 704–706. 10.1111/j.1471-8286.2007.02055.x 21585877

[45] Pritchard JK, Stephens M, Donnelly P. Inference of population structure using multilocus genotype data. Genetics. 2000;155: 945–959. 10835412 10.1093/genetics/155.2.945 PMC1461096

[46] Falush D, Stephens M, Pritchard JK. Inference of population structure using multilocus genotype data: Linked loci and correlated allele frequencies. Genetics. 2003;164: 1567–1587. 12930761 10.1093/genetics/164.4.1567 PMC1462648

[47] Hubisz MJ, Falush D, Stephens M, Pritchard JK. Inferring weak population structure with the assistance of sample group information. Mol Ecol Resour. 2009;9: 1322–1332. 10.1111/j.1755-0998.2009.02591.x 21564903 PMC3518025

[48] Evanno G, Regnaut S, Goudet J. Detecting the number of clusters of individuals using the software STRUCTURE: A simulation study. Mol Ecol. 2005;14: 2611–2620. 10.1111/j.1365-294X.2005.02553.x 15969739

[49] Earl DA, Von Holdt BM. STRUCTURE HARVESTER: A website and program for visualizing STRUCTURE output and implementing the Evanno method. Conserv Genet Resour. 2012;4: 359–361.

[50] Kopelman NM, Mayzel J, Jakobsson M, Rosenberg NA, Mayrose I. Clumpak: A program for identifying clustering modes and packaging population structure inferences across K. Mol Ecol Resour. 2015;15: 1179–1191. 10.1111/1755-0998.12387 25684545 PMC4534335

[51] Jakobsson M, Rosenberg NA. CLUMPP: A cluster matching and permutation program for dealing with label switching and multimodality in analysis of population structure. Bioinformatics. 2007;23: 1801–1806. 10.1093/bioinformatics/btm233 17485429

[52] Rosenberg NA. DISTRUCT: A program for the graphical display of population structure. Mol Ecol Notes. 2004;4: 137–138.

[53] Ronquist F, Teslenko M, Van Der Mark P, Ayres DL, Darling A, Höhna S, et al Mrbayes 3.2: Efficient bayesian phylogenetic inference and model choice across a large model space. Syst Biol. 2012;61: 539–542. 10.1093/sysbio/sys029 22357727 PMC3329765

[54] Lartillot N, Rodrigue N, Stubbs D, Richer J. PhyloBayes MPI: Phylogenetic reconstruction with infinite mixtures of profiles in a parallel environment. Syst Biol. 2013;62: 611–615. 10.1093/sysbio/syt022 23564032

[55] Jobb G, von Haeseler A, Strimmer K. TREEFINDER: a powerful graphical analysis environment for molecular phylogenetics. BMC Evol Biol. 2004;4: 18 10.1186/1471-2148-4-18 15222900 PMC459214

[56] Swofford D.L. PAUP*. Phylogenetic analysis using parsimony (*and other methods). Version 4. Associates S, editor. Sunderland, MA; 1998.

[57] Huelsenbeck JP, Larget B, Alfaro ME. Bayesian phylogenetic model selection using reversible jump Markov chain Monte Carlo. Mol Biol Evol. 2004;21: 1123–1133. 10.1093/molbev/msh123 15034130

[58] Guindon S, Gascuel O. A Simple, Fast, and Accurate Method to Estimate Large Phylogenies by Maximum Likelihood. Syst Biol. 2003;52: 696–704. 14530136 10.1080/10635150390235520

[59] Darriba D, Taboada GL, Doallo R, Posada D. jModelTest 2: more models, new heuristics and parallel computing. Nat Methods. 2012;9: 772–772. 10.1038/nmeth.2109 PMC4594756 22847109

[60] Felsenstein J. PHYLIP (Phylogeny Inference Package) version 3.6. Distributed by the author. Seattle: University of Washington, Department of Genome Sciences.; 2005.

[61] Tamura K, Stecher G, Peterson D, Filipski A, Kumar S. MEGA6: Molecular Evolutionary Genetics Analysis version 6.0. Mol Biol Evol. 2013;30: 2725–2729. 10.1093/molbev/mst197 24132122 PMC3840312

[62] Puillandre N, Lambert A, Brouillet S, Achaz G. ABGD, Automatic Barcode Gap Discovery for primary species delimitation. Mol Ecol. 2012;21: 1864–1877. 10.1111/j.1365-294X.2011.05239.x 21883587

[63] Pons J, Barraclough TG, Gomez-Zurita J, Cardoso A, Duran DP, Hazell S, et al Sequence-Based Species Delimitation for the DNA Taxonomy of Undescribed Insects. Syst Biol. 2006;55: 595–609. 16967577 10.1080/10635150600852011

[64] Monaghan MT, Wild R, Elliot M, Fujisawa T, Balke M, Inward DJG, et al Accelerated species Inventory on Madagascar using coalescent-based models of species Delineation. Syst Biol. 2009;58: 298–311. 10.1093/sysbio/syp027 20525585

[65] Ezard T, Fujisawa T, Barraclough TG. Splits: species’ limits by threshold statistics. R Packag version. 2009;1.

[66] Paradis E, Claude J, Strimmer K. APE: Analyses of phylogenetics and evolution in R language. Bioinformatics. 2004;20: 289–290. 14734327 10.1093/bioinformatics/btg412

[67] Sanderson MJ. Estimating absolute rates of molecular evolution and divergence times: a penalized likelihood approach. Mol Biol Evol. 2002;19: 101–9. 11752195 10.1093/oxfordjournals.molbev.a003974

[68] Nei M, Tajima F, Tateno Y. Accuracy of estimated phylogenetic trees from molecular data. II. Gene frequency data. J Mol Evol. 1983;19: 153–170. 6571220 10.1007/BF02300753

[69] Glückert G. Zur letzten Eistzeit im alpinen und norddeutchen Raum. Geogr Helv. 1987;2: 93–98.

[70] Oberdorfer E. Süddeutsche Pflanzengesellschaften IV, Wälder und Gebüsche. Jena: Gustav Fischer; 1992.

[71] Pfenninger M, Pfenninger A. A new *Trochulus* species from Switzerland (Gastropoda: Pulmonata: Hygromiidae). Arch für Molluskenkd Int J Malacol. 2005;134: 261–269.

[72] Duda M, Kruckenhauser L, Haring E, Sattmann H. Habitat requirements of the pulmonate land snails *Trochulus oreinos oreinos* and *Cylindrus obtusus* endemic to the northern Calcareous Alps, Austria. Eco.mont. 2010;2: 5–12. 10.1553/eco.mont-2-2s5 25729612 PMC4340512

[73] Cruzan MB, Templeton AR. Paleoecology and coalescence: Phylogeographic analysis of hypotheses from the fossil record. Trends in Ecology and Evolution. 2000 pp. 491–496. 11114435 10.1016/s0169-5347(00)01998-4

[74] Stride AH. On the Origin of the Dogger Bank, in the North Sea. Geol Mag. 1959;96: 33.

[75] Proćków M, Kuźnik-Kowalska E, Mackiewicz P. Phenotypic plasticity can explain evolution of sympatric polymorphism in the hairy snail Trochulus hispidus (Linnaeus, 1758). Curr Zool. 10.1093/cz/zow082 PMC5804198 29491999

[76] Duminil J, Caron H, Scotti I, Cazal SO, Petit RJ. Blind population genetics survey of tropical rainforest trees. Mol Ecol. 2006;15: 3505–3513. 10.1111/j.1365-294X.2006.03040.x 17032253

[77] Vanhaecke D, de Leaniz CG, Gajardo G, Young K, Sanzana J, Orellana G, et al DNA barcoding and microsatellites help species delimitation and hybrid identification in endangered galaxiid fishes. PLoS One. 2012;7: e32939 10.1371/journal.pone.0032939 22412956 PMC3295793

[78] Willis SC, Macrander J, Farias IP, Ortí G. Simultaneous delimitation of species and quantification of interspecific hybridization in Amazonian peacock cichlids (genus *Cichla*) using multi-locus data. BMC Evol Biol. 2012;12: 96 10.1186/1471-2148-12-96 22727018 PMC3563476

[79] Shirley MH, Vliet K a, Carr AN, Austin JD. Rigorous approaches to species delimitation have significant implications for African crocodilian systematics and conservation. Proc Biol Sci. 2014;281: 20132483 10.1098/rspb.2013.2483 24335982 PMC3871313

[80] Turini FG, Steinert C, Heubl G, Bringmann G, Kimbadi Lombe B, Mudogo V, et al Microsatellites facilitate species delimitation in Congolese *Ancistrocladus* (Ancistrocladaceae), a genus with pharmacologically potent naphthylisoquinoline alkaloids. Taxon. 2014;63: 329–341.

[81] Dowle EJ, Morgan-Richards M, Trewick SA. Morphological differentiation despite gene flow in an endangered grasshopper. BMC Evol Biol. 2014;14: 216 10.1186/s12862-014-0216-x 25318347 PMC4219001

[82] Supple MA, Papa R, Hines HM, McMillan WO, Counterman BA. Divergence with gene flow across a speciation continuum of *Heliconius* butterflies. BMC Evol Biol. 2015;15: 204 10.1186/s12862-015-0486-y 26403600 PMC4582928

[83] Milá B, Wayne RK, Fitze P, Smith TB. Divergence with gene flow and fine-scale phylogeographical structure in the wedge-billed woodcreeper, *Glyphorynchus spirurus*, a neotropical rainforest bird. Mol Ecol. 2009;18: 2979–2995. 10.1111/j.1365-294X.2009.04251.x 19538341

[84] de León LF, Bermingham E, Podos J, Hendry AP. Divergence with gene flow as facilitated by ecological differences: within-island variation in Darwin’s finches. Philos Trans R Soc Lond B Biol Sci. 2010;365: 1041–1052. 10.1098/rstb.2009.0314 20194167 PMC2830238

[85] Rodríguez-Gómez F, Gutiérrez-Rodríguez C, Ornelas JF. Genetic, phenotypic and ecological divergence with gene flow at the Isthmus of Tehuantepec: The case of the azure-crowned hummingbird (*Amazilia cyanocephala*). J Biogeogr. 2013;40: 1360–1373.

